# Head movement kinematics are altered during balance stability exercises in individuals with vestibular schwannoma

**DOI:** 10.1186/s12984-022-01109-0

**Published:** 2022-11-09

**Authors:** Omid A. Zobeiri, Lin Wang, Jennifer L. Millar, Michael C. Schubert, Kathleen E. Cullen

**Affiliations:** 1 Department of Biomedical Engineering, McGill University, Montreal, QC Canada; 2 Department of Biomedical Engineering, Johns Hopkins University, Baltimore, USA; 3 Department of Physical Medicine and Rehabilitation, Johns Hopkins University School of Medicine, Baltimore, USA; 4 Department of Otolaryngology-Head and Neck Surgery, Johns Hopkins University School of Medicine, Baltimore, USA; 5 Department of Neuroscience, Johns Hopkins University School of Medicine, Baltimore, USA; 6 Kavli Neuroscience Discovery Institute, Johns Hopkins University, Baltimore, USA

**Keywords:** Vestibular schwannoma, Head movements, Balance rehabilitation, Unilateral vestibular deafferentation

## Abstract

**Background:**

Balance stabilization exercises are often prescribed to facilitate compensation in individuals with vestibular schwannoma (VS). However, both the assessment and prescription of these exercises are reliant on clinical observations and expert opinion rather than on quantitative evidence. The aim of this study was to quantify head motion kinematics in individuals with vestibular loss while they performed commonly prescribed balance stability exercises.

**Methods:**

Using inertial measurement units, head movements of individuals with vestibular schwannoma were measured before and after surgical deafferentation and compared with age-matched controls.

**Results:**

We found that individuals with vestibular schwannoma experienced more variable head motion compared to healthy controls both pre- and postoperatively, particularly in absence of visual input, but that there was little difference between preoperative and postoperative kinematic measurements for our vestibular schwannoma group. We further found correlations between head motion kinematic measures during balance exercises, performed in the absence of visual input, and multiple clinical measurements for preoperative VS subjects. Subjects with higher head motion variability also had worse DVA scores, moved more slowly during the Timed up and Go and gait speed tests, and had lower scores on the functional gait assessment. In contrast, we did not find strong correlations between clinical measures and postoperative head kinematics for the same VS subjects.

**Conclusions:**

Our data suggest that further development of such metrics based on the quantification of head motion has merit for the assessment and prescription of balance exercises, as demonstrated by the calculation of a “kinematic score” for identifying the most informative balance exercise (i.e., “Standing on foam eyes closed”).

**Supplementary Information:**

The online version contains supplementary material available at 10.1186/s12984-022-01109-0.

## Introduction

Postural stability, or balance, refers to our ability to keep our center of mass within the limits of our base of support. During everyday activity, the maintenance of balance relies on sensory information from numerous modalities, including the vestibular as well as the visual and somatosensory systems. When peripheral vestibular input to the brain is reduced due to disease, injury, or the aging process, we experience impaired balance in addition to vertigo, gaze instability and significantly increased likelihood of falls and death. For example, in the US population, the prevalence of vestibular dysfunction has been estimated at ~ 35% for adults aged 40 years and older [1] based on balance testing. Due to its high occurrence rate, its impact on quality of life, and the resulting costs associated with individual needs and diminished autonomy—exceeding $20 billion annually [1]—vestibular dysfunction is a major societal burden [2].

The balance dysfunction observed in individuals with vestibular hypofunction is largely a consequence of the decreased efficacy of their vestibulo-spinal reflex (VSR) pathways. Specifically, in everyday life, head motion information is sensed by the vestibular sensory organs located in the temporal bone. These sensory organs include the three orthogonally oriented semicircular canals and the two otolith organs (the utricle and saccule) (reviewed in Ref. [3]). Head motion information is then carried to the brain by vestibular nerve afferents of the VIII cranial nerve. More specifically, vestibular afferents transmit information about our current head motion to the central neurons in the vestibular nuclei that mediate the vestibulo-spinal-reflex pathways [4]. Consistent with relatively direct circuitry projections to the spinal cord, vestibulo-spinal-reflexes generate rapid compensatory balance responses to unexpected head motion. Indeed, vestibular stimulation can evoke compensatory head movements with latencies of 30–40 ms consistent with the known circuitry of vestibulo-spinal pathways [5]. In contrast, visually driven compensations have substantially longer latencies and thus cannot provide such dynamic compensation (reviewed in Refs [6, 7]). Accordingly, individuals with bilaterally absent vestibular function demonstrate impaired balance [8–10].

Due to the marked balance impairment observed in individuals with unilateral or bilateral vestibular loss, current clinical practice guidelines (CPG) recommend balance exercises, under a variety of challenging conditions, as a critical component of rehabilitation efforts [11–15]. Typically, these exercises require subjects to maintain balance under conditions of altered vision (e.g., vision distracted or removed) and/or somatosensory input (e.g., foam or moving surfaces). These exercises often also require changes in the base of support (e.g., Romberg, tandem, single-leg stance) to increase the level of challenge to the subject [11]. However, the recommendation and assessment of balance stabilization rehabilitation exercises are not based on objective criteria. Instead, they follow the subjective assessment of the prescribing clinician [11]. Specifically, to date, no study has objectively quantified head motion kinematics while subjects perform balance stability rehabilitation exercises.

Accordingly, this study was designed to explore the kinematics of the balance stability exercises that are an essential component of vestibular physical therapy in an attempt to isolate critical patterns that will inform treatment. Head motion kinematics were quantified using inertial measurement units (IMUs) in individuals with vestibular schwannoma (VS) during a single session of vestibular rehabilitation balance stability exercises before and 6 weeks after surgical deafferentation. Comparisons were made between the head motion kinematics of preoperative and postoperative VS subjects versus age-matched healthy control subjects. Comparisons were also made between the head movement kinematics of preoperative versus postoperative VS subjects. Additionally, we correlated these kinematic data with standard clinical outcome measures [16–19] to determine whether postoperative head kinematics during balance exercises correlate with preoperative clinical measures. Finally, we addressed whether it was possible to compute a robust “kinematic score” based on head kinematic data obtained from a subset of exercises.

## Methods

### Subjects

We recruited 18 subjects with unilateral vestibular schwannoma that were scheduled for surgical resection. Of these, 9 VS subjects completed all phases of the study (n = 9 males, mean 56.1 ± 15.7 years old, range 24–73 years old), where each VS subject was measured before and 6 weeks after the onset of surgery. The surgical approach for all VS subjects was suboccipital craniotomy and their tumor dimensions per radiology reports ranged from 0.8 to 4.3 (mean 1.9 ± 1.1 cm^3^). We also recruited n = 9 age-matched healthy participants (8 males and 1 female, mean 49.3 ± 15.0 years old, range 24–72 years old) with no history of otologic or neurologic disease. We collected both traditional clinical measures (Additional file 1: Table S1) and kinematics measures at the same time points. Specifically, preoperative measures were collected in an outpatient setting before (mean = 8 ± 13 days) the vestibular schwannoma tumor resection surgery. The postoperative measures were collected at approximately 6 weeks (36–42 days) after the surgery in the same setting as preoperative measures. This study was approved by the Johns Hopkins University Institutional Review Board, and written informed consent was obtained from each participant prior to data collection.

### Clinical measures

#### Dynamic Visual Acuity (DVA)

The DVA test measures the functional outcome of the subjects' vestibulo-ocular reflex (VOR) during active head rotation. DVA was measured using a portable laptop and a motion sensor as developed [20] and validated by Rine et al. [21]. The portable DVA was then normalized in 3992 individuals [22]. We implemented a modified protocol per Millar et al. [23]. Specifically, a Samsung Galaxy Pro tablet (Seoul, South Korea) was used to present the visual stimuli and record the subjects' static and dynamic acuity scores. Static visual acuity was measured first while the subject sat 200 cm from the tablet with their head still. Participants were required to distinguish one letter at a time presented on the tablet. The letter was randomly selected from ten optotypes (capital letters C D H K N O S R V Z). Visual acuity during active sinusoidal head rotations was then measured and scored separately for ipsi and contra-lesional head rotation. Each subject wore a single inertial measurement unit (IMU) (XSENS Technologies, Enschede, Netherlands) attached to a headband. This software generates the visual stimulus once the IMU has detected a head rotation with a velocity greater than 120°/s. The scores were tabulated in the logarithm of the minimal angle resolution (LogMAR). Possible LogMAR scores ranged from − 0.3 to 1.7 (Snellen equivalent of 20/10 to 20/800). Corrected DVA scores were then calculated by subtracting the LogMAR score of static visual acuity from the LogMAR score of ipsilesional and contralesional DVA, respectively.

#### Timed Up and Go (TUG)

The TUG task measured each subject's ability to stand from sitting, walk 3 m and turn 180 degrees before return to seated position. Task performance was scored by measuring the time between when the subject's back exited the chair to when they were seated in the chair again. Each subject completed two TUG trials, turning ipsilesionally, and contralesionally respectively when they passed the obstacle. Scores on the TUG > 11.1 s correlate with reports of falls in persons with vestibular dysfunction [24].

#### Gait speed

The Ten Meter Walk Test (10MWT) measured the subject's self-selected comfortable walking speed over a 10 m distance. The subjects started and stopped at least 2 m beyond the 10 m range to ensure the measured gait speed did not include the acceleration or deceleration phases of the locomotion. Their average gait speed was computed over the 10 m distance.

#### Functional gait assessment (FGA)

The FGA comprises 10 unique walking exercises: (1) Gait on a level surface, (2) Change in gait speed, (3) Gait with horizontal head turns, (4) Gait with vertical head turns, (5) Gait and pivot turn, (6) Step over obstacle, (7) Gait with narrow base of support, (8) Gait with eyes closed, (9) Ambulating backwards, and (10) Steps. An experienced clinician scored each task between 0 and 3 points, with 0 indicating severe impairment and 3 indicating normal performance. FGA scores less than 22 (30 total) are predictive of falls in older adults [25].

### Physiological measures

#### Video Head Impulse Test (vHIT)

The vHIT (ICS Otometrics, Natus Medical Incorporated, Denmark) measures VOR gain (eye velocity/head velocity) during passive head rotation. Subjects were seated 1 m from a stationary visual target, in room light. At least 12 passive head rotations were performed in both directions of three planes parallel to the three pairs of semicircular canals: horizontal, right anterior/left posterior (RALP), and left anterior/right posterior (LARP). Right eye and head velocity were sampled at 220 Hz. vHIT traces were deleted if the eye velocity trace preceded head velocity, if the head velocity was below 100°/s, or if the passive head rotation trace did not match the acceleration profile suggested by the manufacturer. VOR gain values within 0.8–1.2 with standard deviation < 0.12 were considered normal [23, 26, 27].

### Subjective measures

#### Dizziness Handicap Inventory (DHI)

The DHI is a subjective measure, based on a self-report questionnaire, that scores the impact of dizziness or unsteadiness on quality of life. The scale consists of 25 items in functional, emotional, and physical domains, with a total score of 0–100. A higher score corresponds with a worse self-perceived level of disability.

#### Activities-Specific Balance Confidence scale (ABC)

The ABC scale is a self-report measure of balance confidence [28]. The subjective measure consists of 16 self-report items in which subjects rate their confidence of not losing balance while performing various daily activities from 0 (no confidence) to 100 (complete confidence). Previous studies suggested that the ABC score is an accurate indicator of fall risk among individuals with vestibular disorders [29].

#### Headache impact test

The headache impact test measures the impact headaches have on a subject's ability to function in daily life [30]. The scale consists of 6 items in which subjects report how often (e.g., never, rarely, sometimes, very often, or always) headache affects their daily activities. A higher score corresponds with a worse self-perceived level of disability.

#### Beck Anxiety Inventory (BAI)

The BAI is a self-report measure of anxiety with 21 items in which subjects rate their anxiety from: not at all (0), mildly (1), moderately (2), and severely (4) [31]. The total score is the sum of the 21 items, with a score of 0–21 indicating low anxiety, 22–35 indicating moderate anxiety, and scores above 36 indicating a potentially concerning level of anxiety. In the current study, the subjects were instructed to rate their anxiety that is only related to the symptoms caused by the vestibular schwannoma and its resection.

### Kinematic measurements

Subjects were instructed to complete the FGA and 9 balance exercises while their motion was recorded using 6 small (51 mm × 34 mm × 14 mm) motion sensors (Shimmer3 IMU, Shimmer Research, Dublin, Ireland) that were securely and comfortably attached to the subject’s head and body using elastic bands. For each sensor, translational acceleration along the fore-aft, lateral, and vertical axes and angular velocity along the roll, pitch, and yaw planes were recorded. The 6-dimensional data were sampled at 500 Hz and recorded on a built-in microSD card.

To quantify head movements during balance exercises, we computed the following kinematic measures: (i) time duration: the time within 30 s test during which subject could maintain their balance. (ii) Total acceleration standard deviation: the standard deviation of linear acceleration averaged over all three axes (e.g., fore-aft, lateral, and vertical). (iii) Range of motion: the maximum deviation of the motion from the stationary state, computed for all 6 axes of rotation and translation. (iv) Standard deviation: the general deviation from the stationary state during the time that subjects maintained their balance, computed for all 6 axes of rotation and translation.

In addition, we calculated a single kinematic score based on the average weighted linear combination of all kinematic measure as previously described [16]. First, we normalized each of the computed kinematic measures by a linear transformation of mean ± 2SD to obtain a number between 0 and 100 (i.e., normalized mean = 50 and normalized SD = 25). Numbers outside the 0–100 range were then projected to the closest number within this range (i.e., either 0 or 100). The average of three normalized numbers across all selected balance stabilization exercises was then used as the kinematic score.

### Balance exercises

Balance exercises are listed in Table 1. The 9 balance exercises as well as one of the FGA tasks, item 7—Gait with narrow base of support—were examined. These 10 exercises vary in proprioceptive conditions (firm, unstable surfaces, or tandem) and visual conditions (eyes open or eyes closed).

**Table 1:** A list of the 10 balance exercises used in the current study. The exercises vary in proprioceptive conditions and visual conditions

	Task name	Proprioceptive condition	Visual condition
1	Tandem walk forward (FGA)	Tandem	Eyes open
2	Tandem walk forward	Tandem	Eyes open
3	Tandem walk backward	Tandem	Eyes open
4	Tandem stance eyes open	Tandem	Eyes open
5	Tandem stance eyes closed	Tandem	Eyes closed
6	Standing on firm eyes closed	Firm	Eyes closed
7	Standing on foam eyes closed	Unstable surfaces	Eyes closed
8	Standing on foam eyes open	Unstable surfaces	Eyes open
9	Foam cup balance 1 foot	Unstable surfaces	Eyes open
10	Foam cup alternatively foot	Unstable surfaces	Eyes open

For the standing-on-firm-eyes-closed exercise, subjects were required to stand on the ground with their feet together and eyes closed. For the standing-on-foam-eyes-open exercise, subjects were required to stand on a rectangular foam (40 × 32 × 6 cm, 0.8 kg, closed-cell PVC foam, latex free, AIREX®) with their feet together and eyes open. Standing-on-foam-eyes-closed and standing-on-foam-eyes-open exercises were identical except that subjects were required to close their eyes during the former. For the tandem-stance-eyes-open exercise, subjects were required to stand with one foot in front of the other, with the heel of the forward foot touching the toes of backward foot. The tandem-stance-eyes-closed and tandem-stance-eyes-open exercises were identical except that subjects were required to close their eyes during the former. For the foam-cups-balance-1-foot exercise, subjects were required to stand with one foot on the ground and one foot on a foam cup, without crushing the cup. For the foam-cup-alternatively-foot exercise, subjects were required to alternatively tap each foot on the foam cup. For the tandem walk forward/backward exercise, subjects were required to walk forward/backward with arms folded across chest, with the heel of the foot at the front touching the toes of the foot at the back.

During these balance exercises, subjects were positioned with their arms crossed. If a subject moved their arm or feet during the test (detected by the wrist or ankle sensor) to catch themselves, the change in wrist or ankle sensor signal identified during the segmentation process, was an indicator of a “near fall” or “loss of balance”. No subject ever fell to the ground. Subjects were given the opportunity to recover their balance and complete the entire 30 s task, to allow us to capture as much data as possible, rather than discontinuing the test if they took a mis-step or touched the wall after the first few seconds of the test. Gait with narrow base of support (item 7 of functional gait assessment (FGA)) is the same as tandem walk forward except that it requires subjects to walk 10 steps without a time limit.

### Statistics

We performed non-parametric paired sample permutation (re-randomization) tests for all the kinematic measures from the VS subjects (pre- and postoperative) and age-matched healthy controls. Specifically, p-values were computed by (i) pooling data from both groups and then randomly rearranging this pooled data 2000 times, (ii) computing the sample mean for the first N data points of each rearrangement, where N is the number of subjects in first group, (iii) computing the sample mean for the remaining data points (i.e., same size as second group), (iv) computing the difference between the two sample means in (ii) and (iii) above, and (v) finally testing whether the actual difference between the sample means of each group were significantly different.

We also computed the correlation coefficients and p-values of the correlations between kinematic and clinical measures using Pearson correlation. To examine whether trends were consistent across several exercises we examined (i) whether correlations are significant (p < 0.05) for the majority of exercises, and (ii) whether, for all significant correlations the relationship had the same sign (i.e., correlation were consistently positive/negative across exercises). Throughout the text, values are expressed as mean ± 1 SD and significance is reported at p < 0.05. Correction for multiple comparisons was not performed since the goal of this exploratory study was to investigate individuals with unilateral vestibular loss already known to be different from healthy controls based on clinical assessment and performing correction would have exaggerated Type II errors. All data processing and statistical tests were performed using MATLAB (The MathWorks, Inc., Natick, Massachusetts, United States).

## Results

Kinematic measurements are altered in VS subjects both pre‑ and postoperatively compared to healthy controls, particularly in the absence of visual input. No significant improvement at 6 weeks postoperatively.

**Fig. 1:**
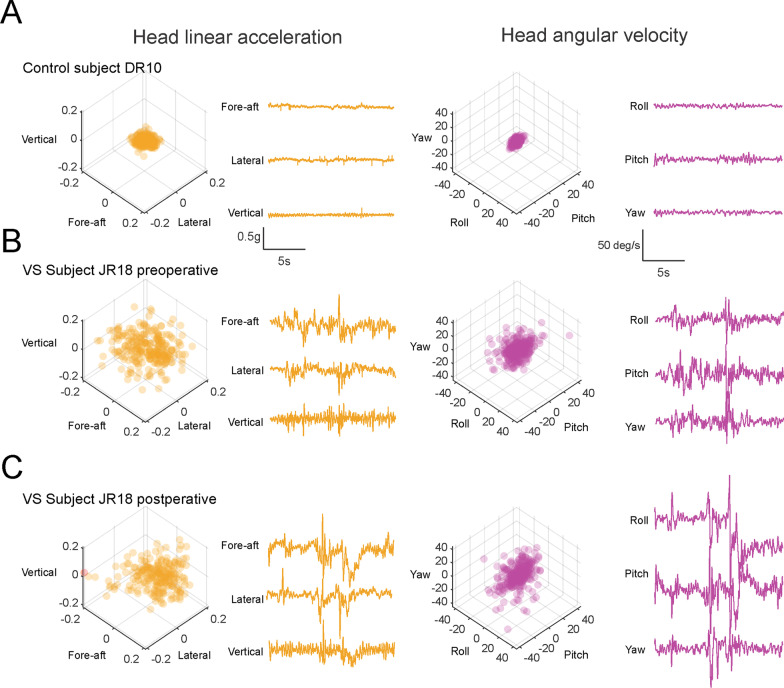
Example data from (**A**) one healthy control and **B** preoperative and **C** postoperative testing from one VS subject in the “Standing-on-foam-eyes-closed” balance stabilization exercise, during which subjects were required to stand on a rectangular foam (15.5 × 12.5 × 2.4 inches) with their feet together and their eyes closed. The gold 3D scatter plots and time series show the head linear acceleration in 3 axes of translation (fore-aft, lateral, and vertical). The purple 3D scatter plots and time series show the head angular velocity in 3 axes of rotation (roll, pitch, and yaw)

We quantified head kinematic data recorded during 10 balance exercises (Table 1) in healthy controls and the same VS subjects across two time points (i.e., preoperative and postoperative). Figure 1 exemplifies head motion from a typical healthy control subject (Fig. 1A), and a typical VS subject pre- and postoperatively (Fig. 1B, C respectively) during one of the more challenging exercises: standing on foam with eyes-closed (Table 1, exercise 7). Comparison across subjects during this example exercise shows that VS subjects (both pre- and postoperatively) displayed a greater range of head motion in all six linear and rotational axes compared to the healthy control subjects (Fig. 1; the gold and purple 3D scatter plots provide a 3D representation of head linear and rotational head motion, respectively), which was indicative of their unstable head motion during this exercise.

**Fig. 2:**
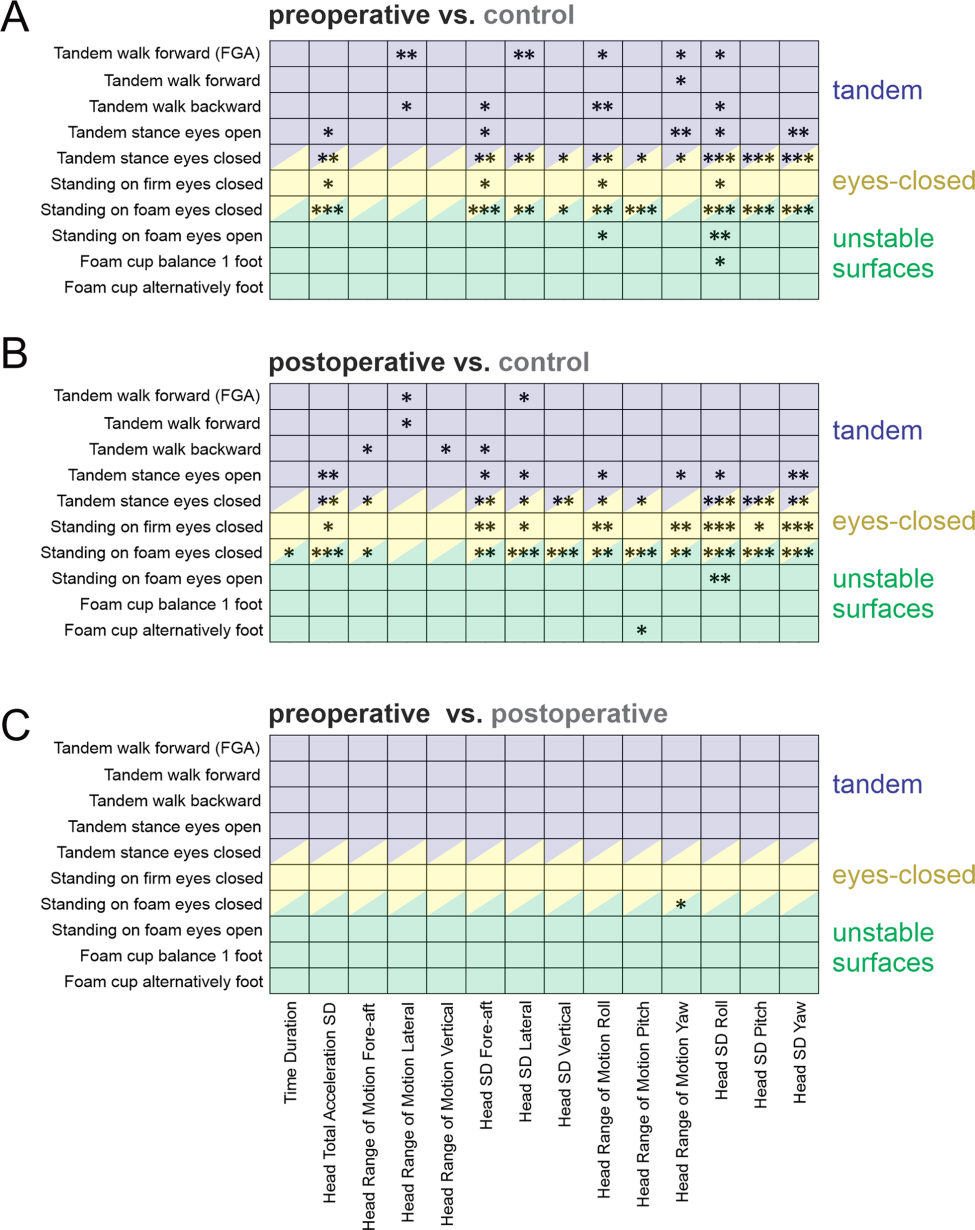
Comparison of kinematic measurements of head motion during balance exercises between **A** preoperative VS subjects and healthy controls, **B** postoperative VS subjects and healthy controls, **C** preoperative and postoperative VS subjects. The kinematic measurements are arranged horizontally. The 10 exercises are arranged vertically and categorized into 3 groups: (i) tandem (purple), (ii) eyes-closed (yellow), and (iii) unstable surface (green). Asterisks indicate significance differences between two groups (*: < 0.05, ** < 0.01, and ***: < 0.001)

Figure 2 illustrates the differences in the measures obtained from our quantification of head motion kinematics (see Methods) during each of the 10 balance exercises, for our populations of control versus VS subjects. The 10 balance exercises were grouped into three categories: (1) ‘tandem stance and tandem walking exercises’ during which the toes of the backward foot touch the heel of the forward foot (purple shaded block), (2) ‘eyes-closed exercises’ during which subjects did not have visual input (yellow shaded block), and (3) ‘exercises that required standing on an unstable surface’ (green shaded block).

Overall, we found that before surgery, measures of movement variability, namely the standard deviation of motion in each of the six dimensions (3 angular velocity and 3 linear acceleration), were the most informative in identifying impairments in the balance performance of VS subjects relative to controls (Fig. 2A). Specifically, VS subjects displayed larger head motion variability in all rotational and linear axes across numerous exercises, with consistent increases in two out of three eyes-closed exercises (i.e., “Tandem stance eyes closed” and “Standing on foam eyes closed”). Figure 2B correspondingly compares the head kinematic measures of postoperative VS versus healthy control subjects using the same approach. Similar to the results of their preoperative testing, VS subjects again displayed larger head motion variability, particularly during the eyes-closed exercises. Additionally, following surgery, VS subjects displayed larger head motion variability during the “standing on firm eyes-closed” exercise, suggesting that the absence of visual input becomes more challenging after the surgery. Interestingly, however, as shown in Fig. 2C, a direct comparison of head movement kinematics before and after surgery revealed little change in the behavior of VS subjects at 6 weeks post‑surgery.

In summary, (i) kinematic measures in VS subjects were altered relative to controls, even before the surgery, due to the impact of their tumor, and (ii) these differences were maintained over at least a 6-week duration from the surgical deafferentation. Table 2 and Additional file 1: Table S2 report the means and standard deviations for the kinematic measures described above, for which there were generally significant differences between groups (Fig. 2); specifically, the standard deviation of head motion in all six axes.

**Table 2:** Standard deviations of head motion in each of the 6 axes for Healthy controls as well as Preoperative and Postoperative VS subjects (Mean ± SD)

	Linear acceleration (mG)			Angular velocity (deg/s)		
	Fore-aft	Lateral	Vertical	Roll	Pitch	Yaw
Tasks	Healthy control					
Tandem walk forward (FGA)	95 ± 40	69 ± 29	63 ± 21	7.0 ± 2.5	10 ± 6.4	6.7 ± 1.9
Tandem walk forward	106 ± 87	68 ± 38	72 ± 44	6.9 ± 3.3	9.0 ± 5.6	6.4 ± 2.4
Tandem walk backward	83 ± 22	77 ± 42	62 ± 24	7.4 ± 3.6	8.2 ± 1.7	6.9 ± 2.3
Tandem stance eyes open	30 ± 12	22 ± 6.9	18 ± 9.7	1.5 ± 0.9	2.3 ± 1.4	1.8 ± 0.6
Tandem stance eyes closed	28 ± 9.9	33 ± 16	21 ± 8.6	2.2 ± 1.1	2.3 ± 1.0	3.0 ± 1.5
Standing on firm eyes closed	23 ± 6.5	18 ± 3.9	14 ± 5.5	0.9 ± 0.2	1.4 ± 0.6	1.2 ± 0.2
Standing on foam eyes closed	27 ± 5.9	23 ± 4.0	18 ± 4.1	1.5 ± 0.4	1.9 ± 0.5	2.0 ± 0.6
Standing on foam eyes open	28 ± 14	20 ± 3.4	15 ± 6.7	1.0 ± 0.3	1.7 ± 1.0	1.7 ± 0.8
Foam cup balance 1 foot	139 ± 72	52 ± 29	54 ± 45	3.6 ± 1.9	8.8 ± 4.5	4.9 ± 2.0
Foam cup alternatively foot	90 ± 38	100 ± 46	59 ± 22	7.6 ± 3.2	8.7 ± 3.2	7.8 ± 3.1
	Preoperative					
Tandem walk forward (FGA)	104 ± 57	106 ± 48	68 ± 34	9.6 ± 4.4	9.7 ± 5.6	8.2 ± 4.2
Tandem walk forward	91 ± 43	93 ± 46	74 ± 41	8.7 ± 4.1	8.8 ± 3.9	7.7 ± 3.7
Tandem walk backward	101 ± 52	107 ± 54	62 ± 31	10 ± 4.9	9.4 ± 5.1	8.2 ± 3.9
Tandem stance eyes open	50 ± 24	40 ± 28	22 ± 12	3.5 ± 2.3	3.9 ± 2.0	3.8 ± 1.9
Tandem stance eyes closed	63 ± 29	94 ± 56	41 ± 20	8.4 ± 4.7	7.2 ± 4.8	7.6 ± 3.4
Standing on firm eyes closed	41 ± 23	27 ± 23	15 ± 9.6	2.1 ± 2.0	2.8 ± 2.4	2.1 ± 1.6
Standing on foam eyes closed	55 ± 34	42 ± 21	34 ± 27	3.8 ± 1.7	4.4 ± 2.4	3.7 ± 1.4
Standing on foam eyes open	32 ± 12	25 ± 15	15 ± 6.6	2.2 ± 1.3	2.4 ± 1.1	2.1 ± 0.8
Foam cup balance 1 foot	93 ± 73	86 ± 53	46 ± 28	7.0 ± 4.2	7.0 ± 3.6	6.1 ± 2.6
Foam cup alternatively foot	74 ± 29	111 ± 28	53 ± 11	9.6 ± 2.7	7.7 ± 2.3	8.5 ± 2.7
	Postoperative					
Tandem walk forward (FGA)	81 ± 48	95 ± 63	66 ± 44	8.7 ± 6.1	6.8 ± 4.2	6.5 ± 4.3
Tandem walk forward	94 ± 64	84 ± 53	67 ± 45	7.6 ± 4.9	7.3 ± 5.0	6.5 ± 4.0
Tandem walk backward	86 ± 51	89 ± 59	65 ± 44	8.2 ± 5.3	7.1 ± 4.3	6.8 ± 4.3
Tandem stance eyes open	67 ± 54	40 ± 24	23 ± 12	3.5 ± 2.3	3.0 ± 1.8	3.5 ± 1.7
Tandem stance eyes closed	73 ± 44	66 ± 39	46 ± 29	5.9 ± 2.4	6.4 ± 2.8	6.3 ± 2.5
Standing on firm eyes closed	39 ± 15	26 ± 9.4	16 ± 7.6	1.8 ± 0.7	2.6 ± 1.3	2.2 ± 0.7
Standing on foam eyes closed	65 ± 42	56 ± 23	38 ± 22	4.8 ± 2.8	6.2 ± 3.3	6.0 ± 3.3
Standing on foam eyes open	59 ± 50	23 ± 9.2	25 ± 23	1.9 ± 0.8	3.1 ± 2.2	2.6 ± 1.4
Foam cup balance 1 foot	92 ± 55	74 ± 42	48 ± 22	6.2 ± 3.8	6.7 ± 3.3	5.8 ± 2.3
Foam cup alternatively foot	65 ± 14	112 ± 39	56 ± 13	8.4 ± 2.9	6.3 ± 0.9	8.4 ± 1.9

### Preoperative head motion during eyes-closed exercises correlates with multiple clinical measurements

**Fig. 3:**
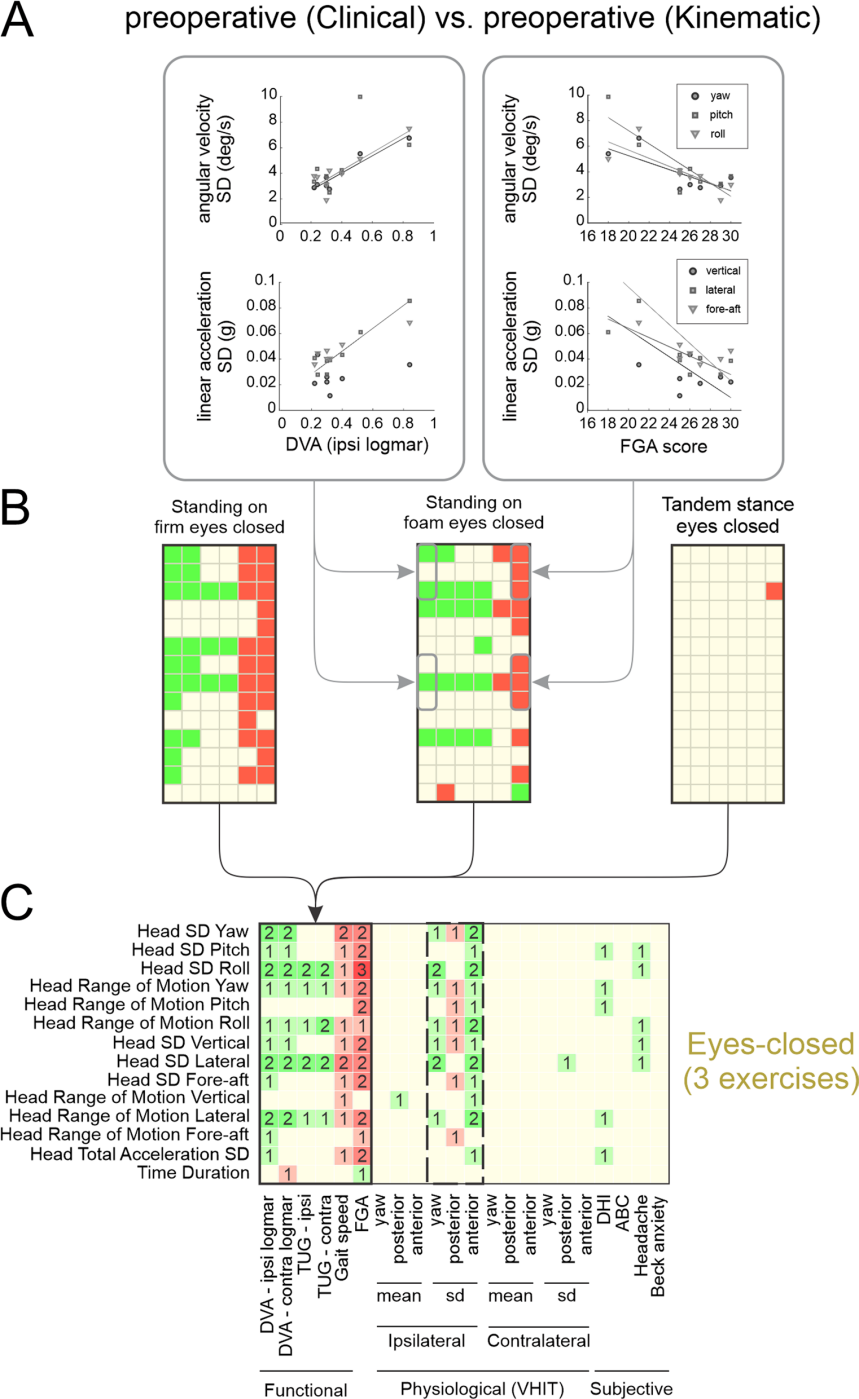
Correlations between preoperative kinematic measurements and pre-surgery clinical measurements. **A** Example of the correlation between standard deviation of the head motion in all 6 axes and functional clinical measures. Left: positive correlation with DVA (ipsi). Right: negative correlation with FGA score. **B** Correlation map between kinematic measures (vertical axis) and functional clinical measures (horizontal axis) for three eyes-closed balance exercises, separately. Green squares indicate positive correlations and red squares indicate negative correlations. **C** Correlation map between kinematic and clinical measures for all three eyes-closed exercises. Brightness and number in the square indicate the number of exercises (1–3) showing a significant correlation (p < 0.05)

We next asked whether there was any relationship between preoperative head kinematic measures during balance tasks and clinical measures. Figure 3A illustrates the significant correlations establishing that preoperative VS subjects**,** who displayed higher variability in angular head velocity (top panels) and linear head acceleration (bottom panels), also have worse ipsilateral DVA scores (r = 0.95, 0.88, and 0.95 for correlations with the standard deviation in lateral, roll, and yaw axes of the head, respectively). Likewise, their FGA scores were lower (r = − 0.84, − 0.75, − 0.75, − 0.78, − 0.85, and − 0.75 for correlations with the standard deviation in fore-aft, lateral, vertical, roll, pitch, and yaw axes, respectively). Additional file 1: Tables S3–12 show the correlation coefficients for all preoperative clinical vs. kinematic measures across each of the 10 balance exercises, with the significant correlations highlighted. Note that red versus green highlights indicate negative versus positive correlations, respectively.

Figure 3B summarizes the relationships between all preoperative kinematic (vertical axis) vs. functional clinical (horizontal axis) measures, for each of the three eyes closed balance exercises. As can be seen, correlations between preoperative head kinematic measures and clinical measures were significant in two out of three eye-closed exercises (i.e., “Standing on firm eyes closed” and “Standing on foam eyes closed”, Fig. 3B, left and middle). Notably, no consistent correlations were found during the “Tandem stance eyes closed” exercise (Fig. 3B, right). Specifically, measures of variability in all 6 axes of motion were positively correlated with DVA and TUG measures (Fig. 3B, green rectangles), and negatively correlated with Gait speed and FGA measures (Fig. 3B, red rectangles). Figure 3C illustrates a combined summary of the relationships between all preoperative kinematic (vertical axis) vs. all clinical measures (i.e., functional, physiological, and subjective measures), during eyes closed balance exercises. Significant relationships were also generally found between preoperative head kinematic measures and clinical physiological measures. For example, head movement kinematic measures displayed significant correlations with the vestibulo-ocular reflex (VOR) gains in ipsilateral yaw and anterior planes (Fig. 3C, dashed rectangle), with more head motion variability positively correlated with more variable VOR gains in yaw and anterior planes. We similarly assessed whether there were any significant relationships between kinematic and clinical measurements during the other type of balance exercises (i.e., tandem and unstable surface), however did not find any consistent correlations (Additional file 1: Fig. S3).

### Preoperative and postoperative clinical measures cannot predict postoperative kinematic measures

**Fig. 4:**
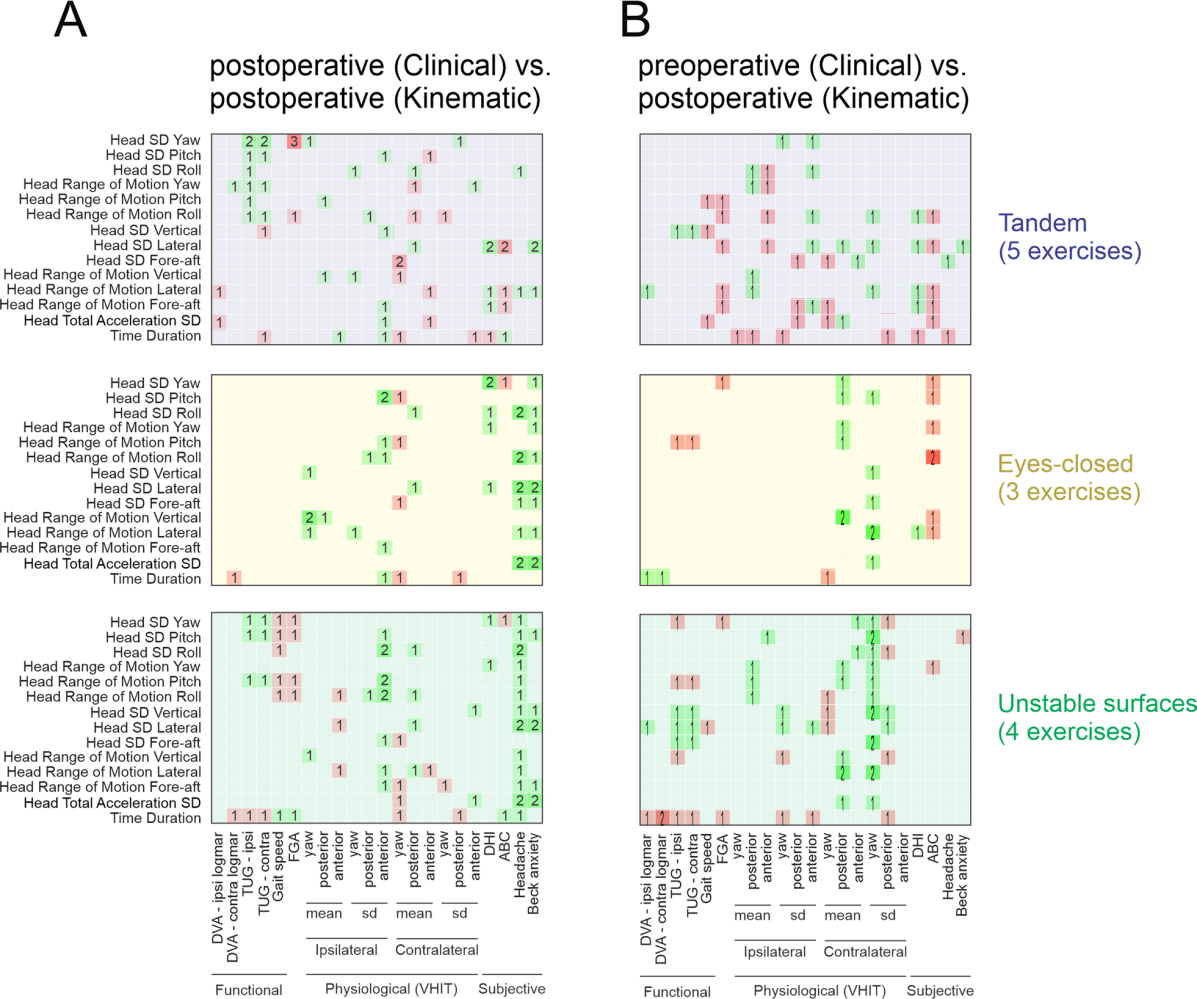
Correlations between postoperative kinematic measurements and **A** post- and **B** preoperative clinical measurements for top: tandem, middle: eyes-closed, and bottom: unstable surface balance exercises. Green squares indicate positive correlations and red squares indicate negative correlations. Brightness and number in the square indicate the number of exercises showing a significant correlation (p < 0.05)

Next, we asked whether there was a relationship between the preoperative and/or postoperative clinical measures of VS subjects and postoperative head kinematics. First, we assessed whether the relationships between kinematic and clinical measures observed in our VS subjects before surgery (Fig. 3) were also observed postoperatively. Figure 4A illustrates the significance of the correlations between postoperative measurements across exercises. Interestingly, unlike what was observed above for the preoperative state, we did not find consistent significant correlation in any group of exercises. Additional file 1: Tables S13–22 show the correlation coefficients for all postoperative clinical vs. kinematic measures across each of the 10 balance exercises, with the significant correlations highlighted. Second, we asked whether we could leverage our quantification of clinical and physiological measures, in the preoperative state to predict the head kinematics of VS subjects, during the balance exercises after the surgery. This analysis is shown in Fig. 4B, which again reports the number of significant correlations across exercises. Comparison between preoperative clinical and physiological measurements and postoperative kinematic measures, however, revealed no consistent relationship. Thus, neither clinical nor physiological measures in the preoperative state predicted the head kinematic measures of VS subjects during the balance exercises after surgery. Additional file 1: Tables S23–32 show the correlation coefficients for all preoperative clinical vs. postoperative kinematic measures across each of the 10 balance exercises, with the significant correlations highlighted.

### Quantifying the global change in head kinematics in VS subjects before and after vestibular neurectomy based on the most informative kinematic parameters

**Fig. 5:**
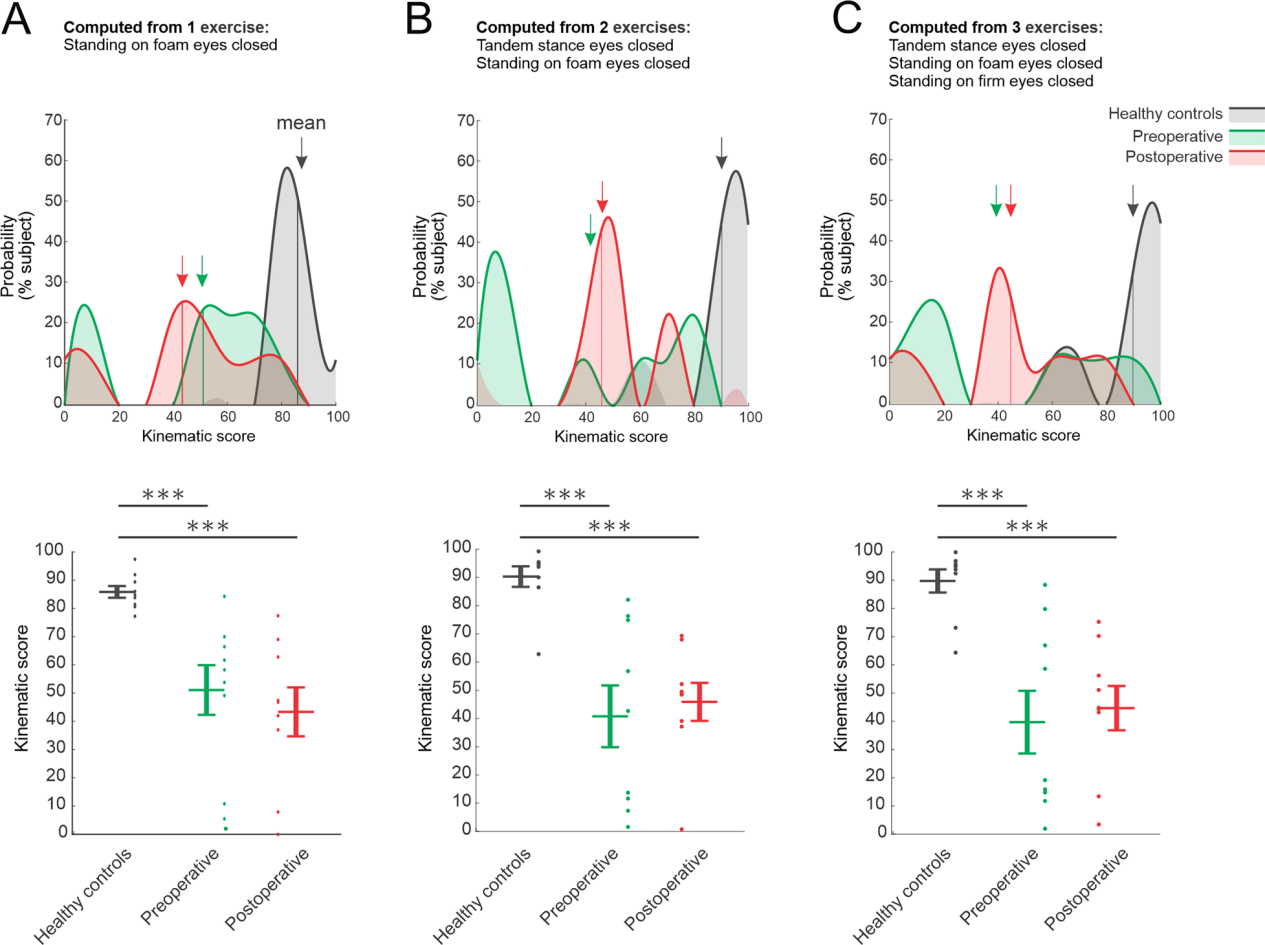
Comparison kinematic scores computed for **A** the 1 most informative balance stabilization exercises ("Standing on foam eyes closed"), **B** 2 balance stabilization exercises (“Tandem stance eyes closed” and " Standing on foam eyes closed"), and **C** across all 3 eyes-closed exercises. **A**–**C** Top: Probability distributions of the kinematic scores computed for healthy controls (black), preoperative (green), and postoperative VS subjects (red). Arrows indicate the average values. Bottom: Comparison of the kinematic scores of healthy controls versus unilateral vestibular VS subjects. Vertical lines correspond to mean ± SEM of the kinematic score for each group, while the kinematic score for individual subjects is illustrated as points. Asterisks denote significant difference between healthy controls and VS subjects (***p < 0.001)

Finally, we determined whether it was feasible to collapse the wide range of kinematic measures we made across exercises into a global kinematic score. To this end, we computed a single score (see Methods) based on the standard deviation of head motion in all 6 axes (i.e., consistently displaying significant differences relative to healthy controls) (Fig. 2). We compared this score when it was computed for (i) the most informative balance exercise (i.e., that exercise for which we found the most significant differences between VS subjects and healthy controls, “Standing on foam eyes closed”; Fig. 5A), (ii) the 2 most informative exercises (i.e., “Standing on foam eyes closed” and “Tandem stance eyes closed”; Fig. 5B), and (iii) these 2 exercises combined with a third exercise that showed the consistent significant differences between postoperative and healthy controls (i.e., “Standing on foam eyes closed”, “Tandem stance eyes closed”, and “Standing on firm eyes closed”; Fig. 5C). The computed kinematic score (see Methods) spanning a range from 0 (most altered) to 100 (comparable to healthy controls).

As can be seen, the kinematic score computed for each of the above 3 cases was similar, with healthy controls scoring closest to 100%, followed by preoperative VS subjects, and then postoperative VS subjects. Notably, controls were significantly different than both preoperative (p < 0.001) and postoperative VS subjects (p < 0.001) regardless of the computation. Furthermore, we observed considerable inter-subject variability in the kinematic-based scores computed across our population of preoperative and postoperative VS subjects (Fig. 5; green shaded region), with some individual scores comparable to those of healthy controls (> 50) but also lower individual scores (< 50). Overall, our analysis suggests the utility of computing an individual’s “kinematic score” based on head kinematic data obtained from a subset of exercises. Also, these results highlight the potential utility of focusing on a single stabilization exercise (e.g., “Standing on foam eyes closed”) in the assessment of head kinematics to develop an informative “kinematic score” for the objective quantification of balance impairments. Further algorithm development and testing utilizing a larger dataset will be required to test this possibility.

## Discussion

In this study, we investigated whether head motion kinematics are altered because of peripheral vestibular loss during balance exercises. To do this, we quantified head motion kinematics in VS subjects before and 6 weeks after unilateral vestibular nerve deafferentation, using an accessible and inexpensive method compared to more expensive posturography. Our analysis of head motion revealed several interesting findings that have potential clinical implications. First, VS subjects showed altered kinematics relative to age-matched control subjects before the surgery. Interestingly, VS subjects experienced more head motion variability relative to healthy controls both before and after surgery, particularly during exercises that were performed in the absence of visual input. Second, the increased variability observed before surgery remained postoperatively for the same exercises. Third, in preoperative VS subjects, changes in head kinematics were predictive of multiple clinical test scores (e.g., TUG, DVA, FGA). Finally, we establish that a “kinematic score” based on head kinematic data obtained during the single most informative balance exercises (i.e., “standing on foam eyes closed”) alone can provide valuable information for evaluating VS subjects. Taken together our findings demonstrate the robustness of the general approach. We suggest that the further development of such metrics during a subset of the standard balance exercises recommended by clinical practice guidelines (CPG) is interesting direction for assessment and prescription—that can ultimately be used to provide real time feedback via kinematic data to both the clinician and patient during the rehabilitation process.

### IMU measures of postural stability and implications for vestibular rehabilitation

Posturography requiring a force plate to evaluate the body’s postural stability has long been the gold standard for the assessment of balance (see, for example, the Equitest developed by Neurocom Inc) [32]. Using this method, a clinician can effectively measure the trajectory (postural sway) of the subject’s center of pressure (COP) over time. More recently, developments in inertial measurement units (IMUs) that sense 6-dimensional motion have facilitated the ability to assess balance in subjects [33, 34]. IMUs provide a less expensive option that is also lightweight and portable making it more practical to use in clinical settings as well as other environments. As a result, over the past several years numerous studies have taken advantage of an IMU-based approach to assessing postural stability in healthy as well as patient subjects (e.g., Refs. [35–47]). The application of computational methods to IMU-based data sets has demonstrated that this method can provide a clinician with a comparable ability to differentiate between individuals with vestibular loss and healthy controls as force platform posturography [48]. Furthermore, machine learning algorithms, which extract important features from IMU data, can be trained to produce estimates of balance performance that are as accurate as those of expert examiners (i.e., trained physical therapists) [49, 50].

However, while there is increasing evidence to support the use of IMUs to evaluate balance, their use in vestibular rehabilitation in VS or other subject groups is neither common nor generally accepted as a standard of care. This is in part related to an absence of norms for such testing. A recent study quantified the perceived intensity of standing balance exercises in healthy subjects by measuring postural sway with body-mounted IMUs [51]. Yet, prior to our study, there had been no systematic quantification of six-dimensional head movement kinematics during standard balance exercises recommended by the CPG or understanding of whether a particular subset of these exercises/measures were the most informative regarding the objective quantification of balance impairments, and thus potentially most beneficial to these individuals. The current CPG recommends balance exercises, under a variety of challenging conditions, as a critical component of rehabilitation efforts [11–14]. Typically, these exercises require subjects to balance under conditions of altered visual (e.g., vision distracted or removed) and/or somatosensory input (e.g., foam or moving surfaces). They often can require changes in the base of support (e.g., Romberg, tandem, single-leg stance) to increase the level of challenge to the subject [11].

In this context, the present study extends the existing literature. Most notably our findings provide the first normative data set of 6-dimensional head motion kinematics during balance exercises in VS subjects before and after surgery as well as age-matched controls. Quantification of our data set specifically revealed that head movement kinematics can be predictive of clinical measures, notably DVA, TUG, functional gait assessment, and gait speed scores (see Fig. 3). Our results further demonstrate that it is not only feasible (i) to collapse the wide range of measures we made into a global kinematic score that may be useful to clinicians interested to know how their interventions may alter balance behavior before and after tumor resection but also (ii) focus on a single balance stabilization exercise (e.g., “Standing on foam eyes closed”). We speculate that this will be a constructive area for the investigation of further algorithm development and testing. Finally, we emphasize that our kinematic based approach is complementary to traditional clinical assessments that are commonly used to identify individuals with unilateral vestibular loss relative to healthy controls. Further work will be needed to fully understand whether and how additional variables such as tumor size and grade influence kinematic balance measures in VS subjects [52]. Interestingly, Parietti-Winkler et al. recently reported that tumor size was not predictive of short-term postural recovery measured via posturography [53], suggesting that this might also be the case for kinematic balance measures.

### Comparison of changes in head movement kinematics during balance versus gaze stability exercises in vestibular schwannoma subjects

In a recent study, we carried out a parallel analysis in which we quantified head motion kinematics of the same VS subjects as this study during ‘gaze stabilization’ exercises that are also recommended by the CPG [11–16]. A comparison between our present results and this prior study reveals some interesting common trends. First, VS subjects displayed significantly altered head movement kinematics during both balance exercises—performed with the eyes closed—and gaze exercises. In particular, VS subjects’ head movement kinematics were different from those of age-matched control subjects both before and 6 weeks following unilateral vestibular nerve deafferentation. Second, a comparison of VS subjects’ head movement kinematics before and after vestibular nerve deafferentation revealed analogous results for both balance (present study) and gaze stabilization exercise [16]. Specifically, while VS subjects’ head movements were altered to those of control subjects, they remained unchanged when compared before versus after surgery. Finally, comparison of clinical measures and head movement kinematics revealed that most functional (e.g., DVA, TUG time, and gait speed), and some specific physiological (i.e., variability of VOR gain for horizontal and anterior planes) clinical measures correlated with VS subjects’ head movement kinematics—at a given time point (i.e., both measures were made either prior to or following surgery).

However, there are also some significant differences between the results of these two studies. Wang et al. found that head movement kinematics during gaze stabilization exercises following surgery were not only a good predictor of clinical outcomes, but also that preoperative clinical measures could actually predict postoperative head kinematics [16]. This exciting result led to the proposal that the VS subjects’ vestibular impairment before the surgery was predictive of their head movement kinematics after the vestibular nerve deafferentation, and that rehabilitation training prior to the surgery may be advantageous. In contrast, in the present study, while the quantification of head movement kinematics during balance exercises following surgery was a good predictor of clinical outcomes, preoperative clinical measures did not predict postoperative head kinematics.

We speculate that preoperative clinical measures were not predictive of postoperative head kinematics during balance exercises (as opposed to gaze stabilization exercise) because VS subjects were able to adopt a wider range of compensation strategies for their balance vs. gaze impairments. Following acute peripheral vestibular loss, improvements in balance are largely mediated by central compensatory mechanisms in VSR pathways [55] that up-weight the brain’s reliance on extra-vestibular sensory cues including vision and proprioception [6, 7]. The VSR is predominately mediated by indirect pathways including relay structures such as the interstitial nucleus of Cajal and reticular formation, in addition to direct projections from the vestibular nuclei to the neck motoneurons (reviewed in Ref. [4, 57]). Changes in balance strategy can occur to integrate longer latency visual inputs [58, 59] with centrally programmed predictive motor commands within the indirect VSR pathways [54].

In contrast, the VOR pathway underlying stable gaze is predominately mediated by a direct three-neuron arc; vestibular afferents project to the vestibular nuclei, which in turn project to the motoneurons that activate the eye muscles. Thus while extra-vestibular proprioceptive and motor inputs are upweighted in VOR pathways following peripheral vestibular loss [56], visual inputs are too slow to effectively contribute to the direct pathway ensuring stable gaze. Indeed, consistent with this idea, our present findings show that—both before and after surgery—VS subjects demonstrated poorer stability (i.e., larger head accelerations and velocities) as compared to controls during balance stabilization exercises; even when performing exercises where visual cues were present (i.e., “Tandem stance eyes open”).

### Conclusions and implications

Our results have immediate clinical implications in that they provide evidence for the clinical benefit of IMU-captured head kinematic data. For example, the head kinematics of preoperative VS subjects are predictive of their clinical test scores, and the quantification of altered movement patterns to obtain a single “kinematic score” offers a novel means for identifying VS subjects. While the use of such a “kinematic score” will require further algorithm development, it has the potential to improve clinical efficiency. Furthermore, compared to more expensive posturography, our approach provides a relatively accessible and inexpensive method for subject testing. Notably, kinematic data, in general, characterizes the status of a subject’s impairment and progress in an objective manner that is not otherwise visible to clinicians that can ultimately be used to provide real-time feedback. Such real-time feedback is likely to improve the efficiency of the rehabilitation effort, as well as provide new insight for understanding how individuals adapt their balance strategies not only in loss of sensory afference but also in the restoration/augmentation of sensory input.

## Supplementary Information

**Additional file 1.** Additional Tables and Figure.

## Data Availability

The data that support the findings of this study are available from the corresponding author, K.E.C, upon reasonable request.

## Abbreviations

ABC: Activities-Specific Balance Confidence scale
BAI: Beck Anxiety Inventory
CPG: Clinical practice guidelines
DHI: Dizziness Handicap Inventory
DVA: Dynamic Visual Acuity
FGA: Functional gait assessment
IMU: Inertial measurement unit
LARP: Left anterior/right posterior
RALP: Right anterior/left posterior
RMS: Root mean square
SD: Standard deviation
TUG: Timed Up and Go
vHIT: Video Head Impulse Test
VOR: Vestibulo–Ocular Reflex
VS: Vestibular Schwannoma
