# Supplementary material for: Head movement kinematics are altered during balance stability exercises in individuals with vestibular schwannoma
Source: J Neuroeng Rehabil. 2022 Nov 9;19:120. doi: 10.1186/s12984-022-01109-0 (PMC9648040; doi:10.1186/s12984-022-01109-0)
Supplement: Supplementary file 1 — Additional file 1. Additional Tables and Figure. [file 12984_2022_1109_MOESM1_ESM.docx]

| Postoperative | 0.48±0.21 | 0.31±0.13 | 25.2±6.6 | 9.51±5.9 | 9.04±5.3 | 1.26±0.3 | 0.48±0.28 | 0.36±0.21 | 0.45±0.16 | 0.63±0.26 | 0.70±0.38 | 0.53±0.19 | 33.0±24.7 | 76.1±26.6 | 51.3±10.0 | 6.9±4.3 |
| --- | --- | --- | --- | --- | --- | --- | --- | --- | --- | --- | --- | --- | --- | --- | --- | --- |
| Preoperative | 0.36±0.21 | 0.31±0.13 | 25.6±3.9 | 7.69±3.0 | 7.54±2.8 | 1.38±0.3 | 0.78±0.27 | 0.61±0.18 | 0.71±24 | 0.91±0.15 | 0.79±23 | 0.62±0.24 | 20.4±21.8 | 86±17.7 | 50.2±7.4 | 7.6±6.0 |
| Healthy control | 0.23±0.07 | 0.21±0.09 | 28.9±0.4 | 6.32±1.3 | 6.41±1.1 | 1.53±0.2 | 0.99±0.08 | 0.82±0.09 | 0.80±0.34 | 0.93±0.04 | 0.85±0.19 | 0.75±0.12 | 0.9±2.7 | 94.4±7.4 | 37.0±15.8 | 2.8±3.1 |
|  | ipsilateral | contralateral | FGA score | Ipsilateral (s) | Contralateral (s) | Gait Speed (m/s) | Horizontal | Posterior | Anterior | Horizontal | Posterior | Anterior | DHI | ABC | HIT | Beck |
|  | DVA | |  | TUG | |  | ipsilateral | | | contralateral | | |  |  |  |  |
|  | Functional | | | | | | Physiological (VHIT) | | | | | | Subjective | | | |

**Table 1- table supplement –** Clinical measures (functional, physiologic, subjective) collected from preoperative and postoperative patients, and healthy controls.

Table 2 –table supplement

**Table 2- table supplement -** Range of head motion in 6 axes for Healthy controls as well as Preoperative and Postoperative patients (Mean±SD).

|  | Linear Acceleration (mG) | | | Angular Velocity (deg/s) | | |
| --- | --- | --- | --- | --- | --- | --- |
|  | Fore-aft | Lateral | Vertical | Roll | Pitch | Yaw |
| Tasks | Healthy control | | | | | |
| Tandem walk forward (FGA) | 601±232 | 489±160 | 635±394 | 43±17 | 83±77 | 39±12 |
| Tandem walk forward | 657±361 | 522±255 | 785±522 | 46±20 | 80±66 | 42±19 |
| Tandem walk backward | 596±200 | 611±316 | 663±245 | 50±23 | 61±19 | 52±17 |
| Tandem stance eyes open | 319±60 | 295±84 | 314±263 | 15±14 | 23±18 | 15±8.2 |
| Tandem stance eyes closed | 291±114 | 386±258 | 359±244 | 19±16 | 19±15 | 24±21 |
| Standing on firm eyes closed | 288±89 | 235±92 | 267±107 | 7.5±2.0 | 13±8.5 | 9.3±2.4 |
| Standing on foam eyes closed | 262±94 | 271±84 | 261±119 | 12±4.6 | 14±4.5 | 17±10 |
| Standing on foam eyes open | 290±136 | 245±108 | 229±122 | 9.6±5.9 | 20±14 | 21±26 |
| Foam cup balance 1 foot | 819±288 | 447±203 | 550±254 | 41±31 | 109±76 | 56±32 |
| Foam cup alternatively foot | 650±269 | 569±201 | 628±354 | 54±21 | 94±60 | 72±37 |
|  | Preoperative | | | | | |
| Tandem walk forward (FGA) | 675±358 | 757±356 | 785±481 | 66±34 | 70±42 | 57±32 |
| Tandem walk forward | 749±409 | 758±437 | 850±542 | 58±30 | 77±41 | 62±36 |
| Tandem walk backward | 695±353 | 906±396 | 769±555 | 77±36 | 75±44 | 70±40 |
| Tandem stance eyes open | 411±161 | 375±171 | 288±125 | 30±18 | 43±30 | 32±19 |
| Tandem stance eyes closed | 449±214 | 654±372 | 402±293 | 55±30 | 47±27 | 53±20 |
| Standing on firm eyes closed | 340±163 | 252±127 | 235±114 | 18±16 | 24±18 | 17±14 |
| Standing on foam eyes closed | 373±175 | 351±130 | 302±152 | 29±13 | 29±14 | 26±9.1 |
| Standing on foam eyes open | 322±112 | 280±96 | 264±176 | 22±12 | 29±23 | 19±7.6 |
| Foam cup balance 1 foot | 604±317 | 646±339 | 630±350 | 60±34 | 69±38 | 54±32 |
| Foam cup alternatively foot | 569±265 | 741±217 | 608±275 | 69±19 | 69±40 | 72±43 |
|  | Postoperative | | | | | |
| Tandem walk forward (FGA) | 493±301 | 658±443 | 724±506 | 55±41 | 48±31 | 45±32 |
| Tandem walk forward | 654±428 | 661±423 | 945±886 | 54±37 | 65±50 | 52±36 |
| Tandem walk backward | 646±396 | 779±530 | 966±772 | 64±46 | 61±39 | 55±33 |
| Tandem stance eyes open | 377±189 | 354±188 | 328±202 | 33±20 | 24±15 | 27±12 |
| Tandem stance eyes closed | 478±199 | 411±149 | 397±207 | 36±17 | 42±20 | 38±16 |
| Standing on firm eyes closed | 322±104 | 257±99 | 257±88 | 16±7.0 | 25±16 | 17±5.9 |
| Standing on foam eyes closed | 456±216 | 413±190 | 334±139 | 35±21 | 47±27 | 44±25 |
| Standing on foam eyes open | 399±233 | 242±91 | 286±114 | 16±8.6 | 34±36 | 24±18 |
| Foam cup balance 1 foot | 664±292 | 596±336 | 554±384 | 46±28 | 68±44 | 47±25 |
| Foam cup alternatively foot | 556±208 | 723±363 | 859±575 | 61±33 | 51±16 | 57±19 |


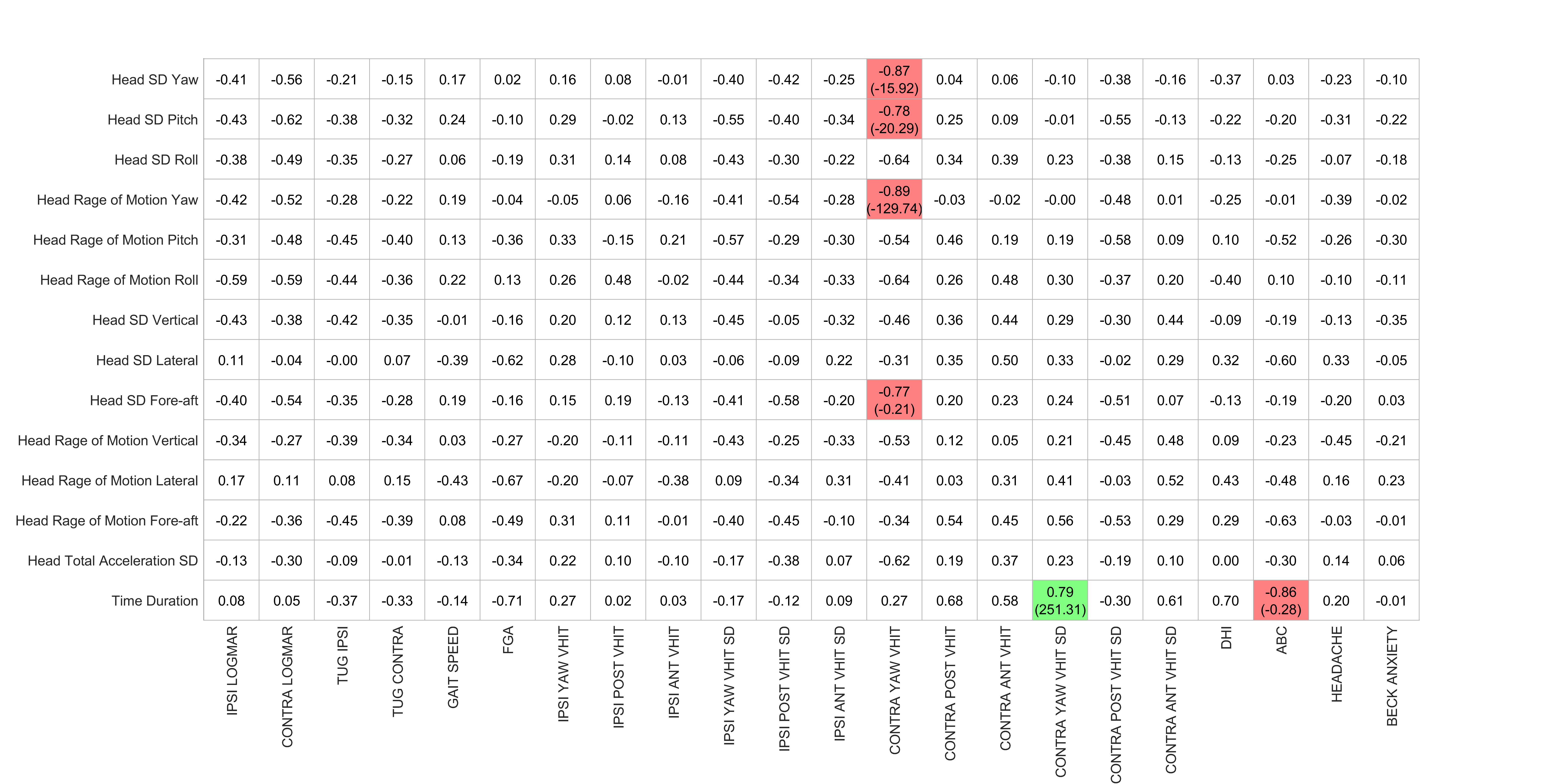


**Table 3- table supplement -** Correlation coefficients (slope) for Task “Tandem walk forward (FGA)” (Preop. Clinical vs. Preop. Kinematics)


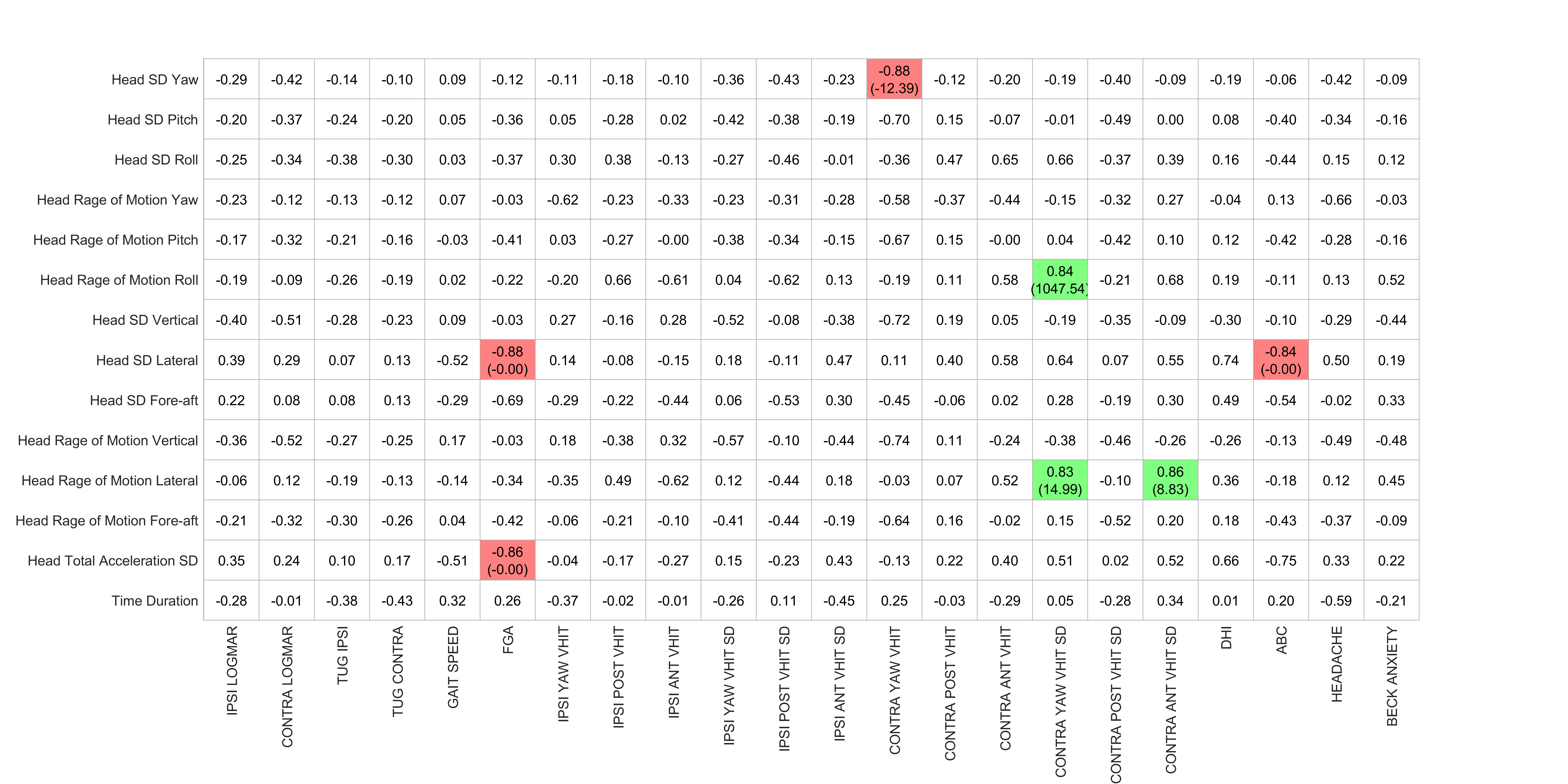


**Table 4- table supplement -** Correlation coefficients (slope) for Task “Tandem walk forward” (Preop. Clinical vs. Preop. Kinematics)


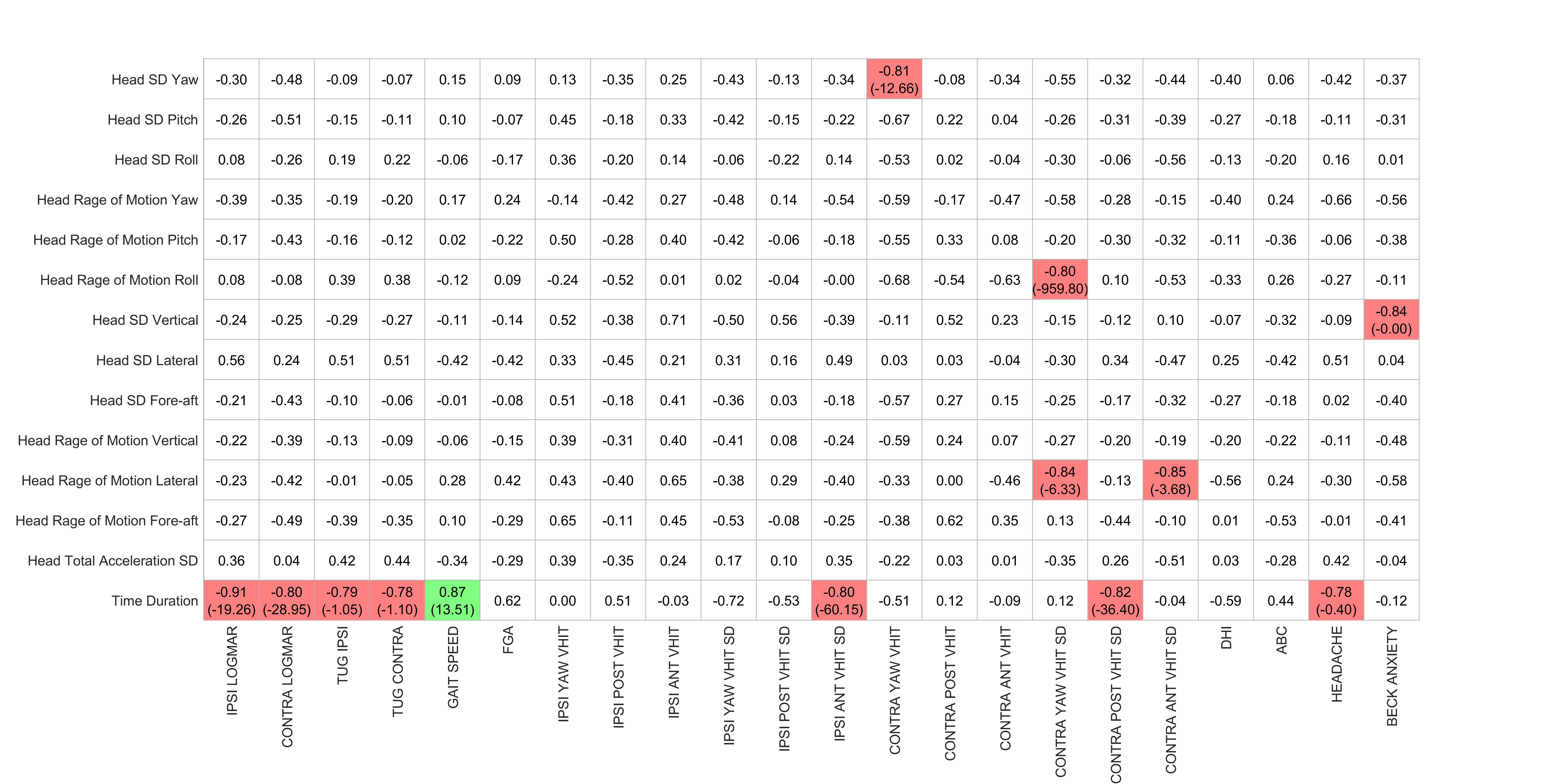


**Table 5- table supplement -** Correlation coefficients (slope) for Task “Tandem walk backward” (Preop. Clinical vs. Preop. Kinematics)


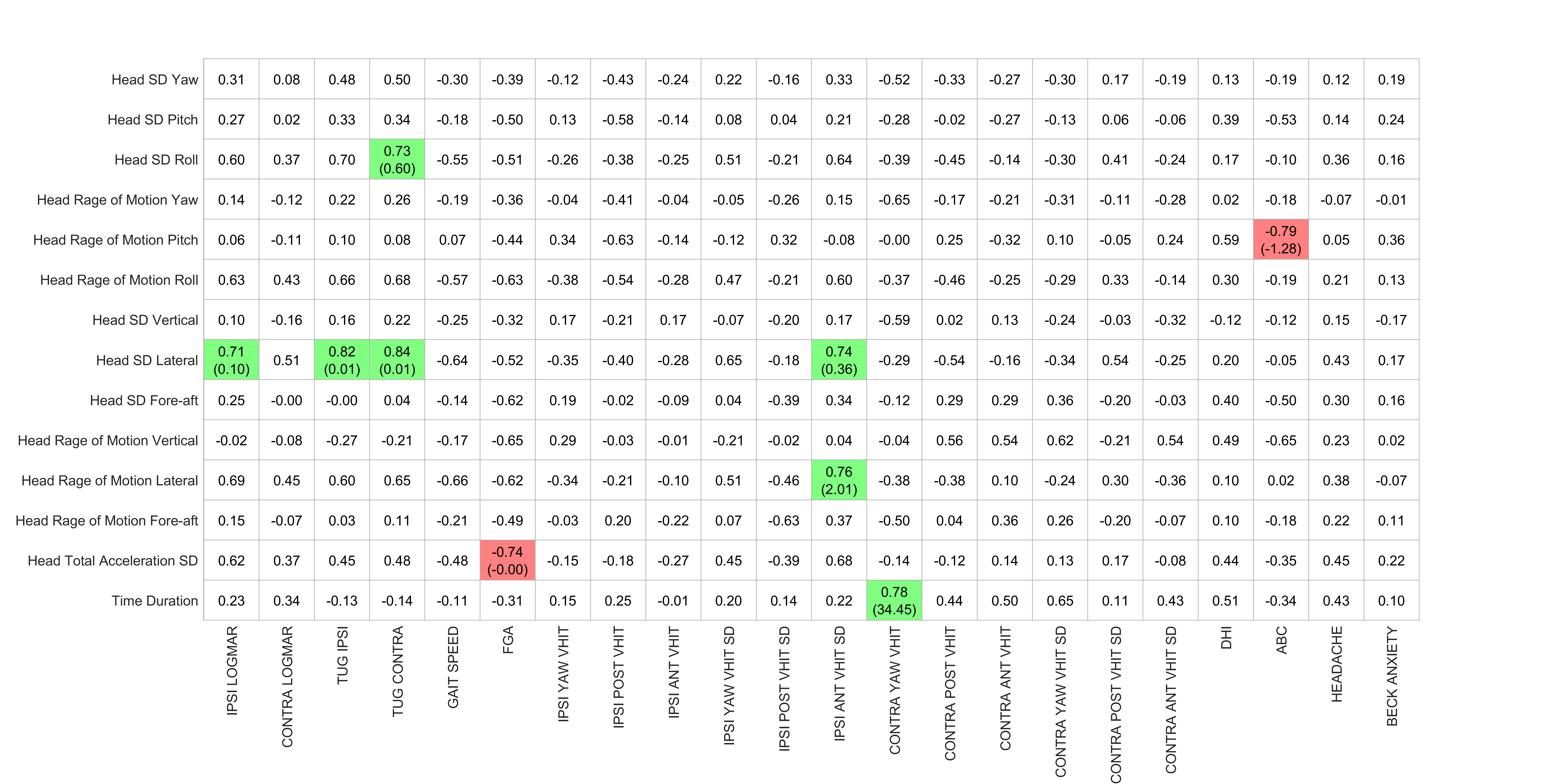


**Table 6- table supplement -** Correlation coefficients (slope) for Task “Tandem stance eyes open” (Preop. Clinical vs. Preop. Kinematics)


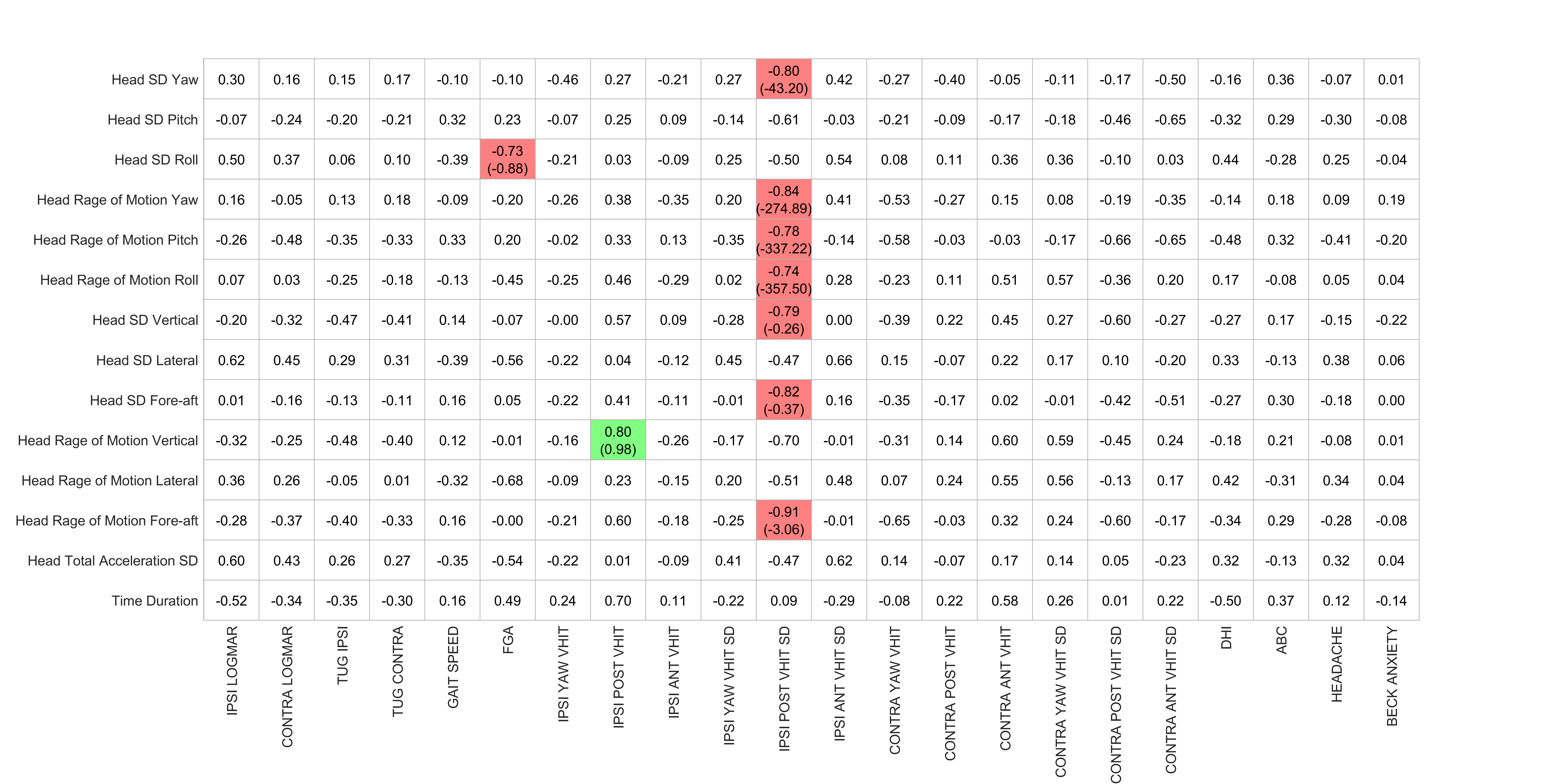


**Table 7- table supplement -** Correlation coefficients (slope) for Task “Tandem stance eyes closed” (Preop. Clinical vs. Preop. Kinematics)

**
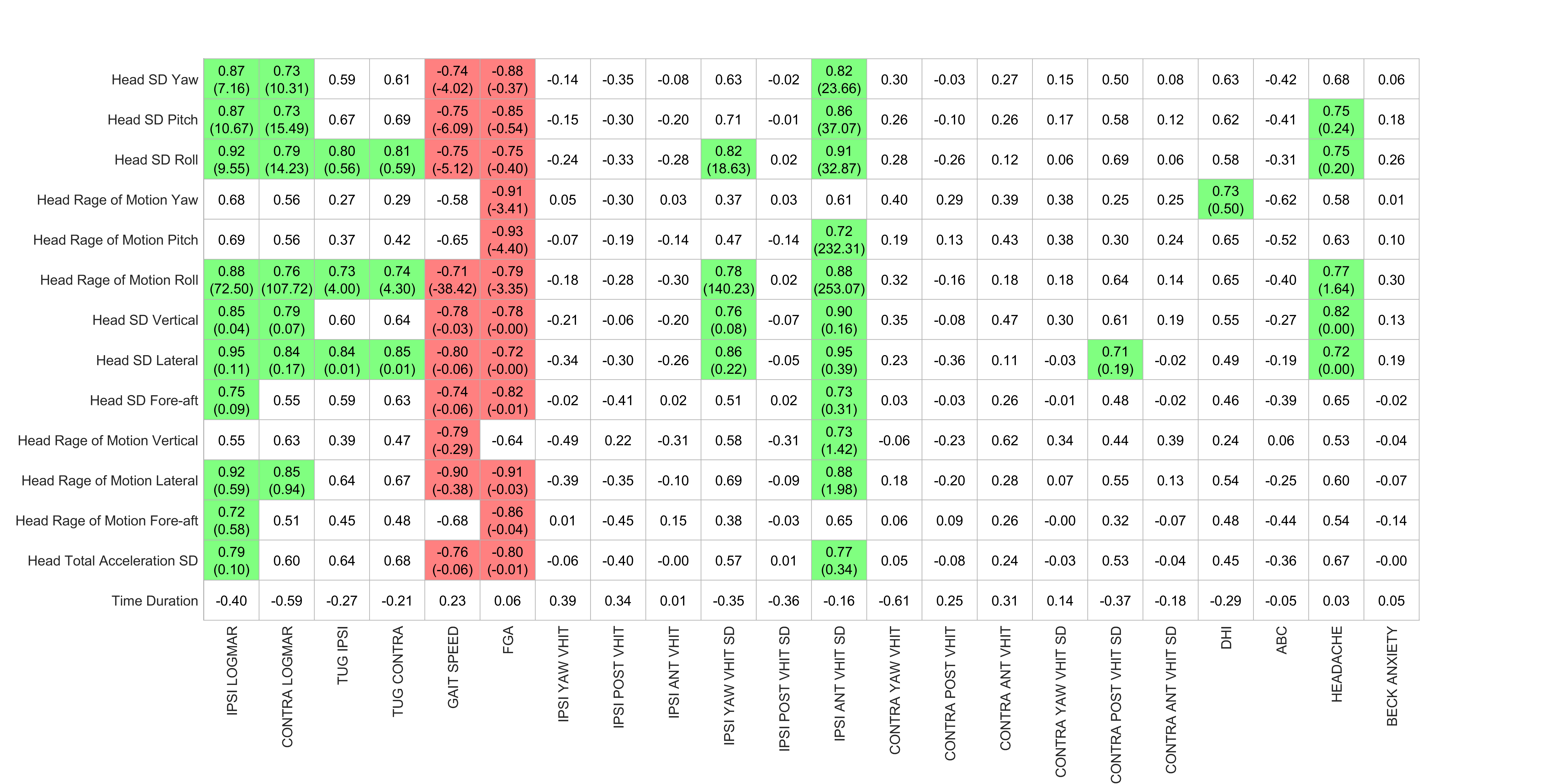
**

**Table 8- table supplement -** Correlation coefficients (slope) for Task “Standing on firm eyes closed” (Preop. Clinical vs. Preop. Kinematics)


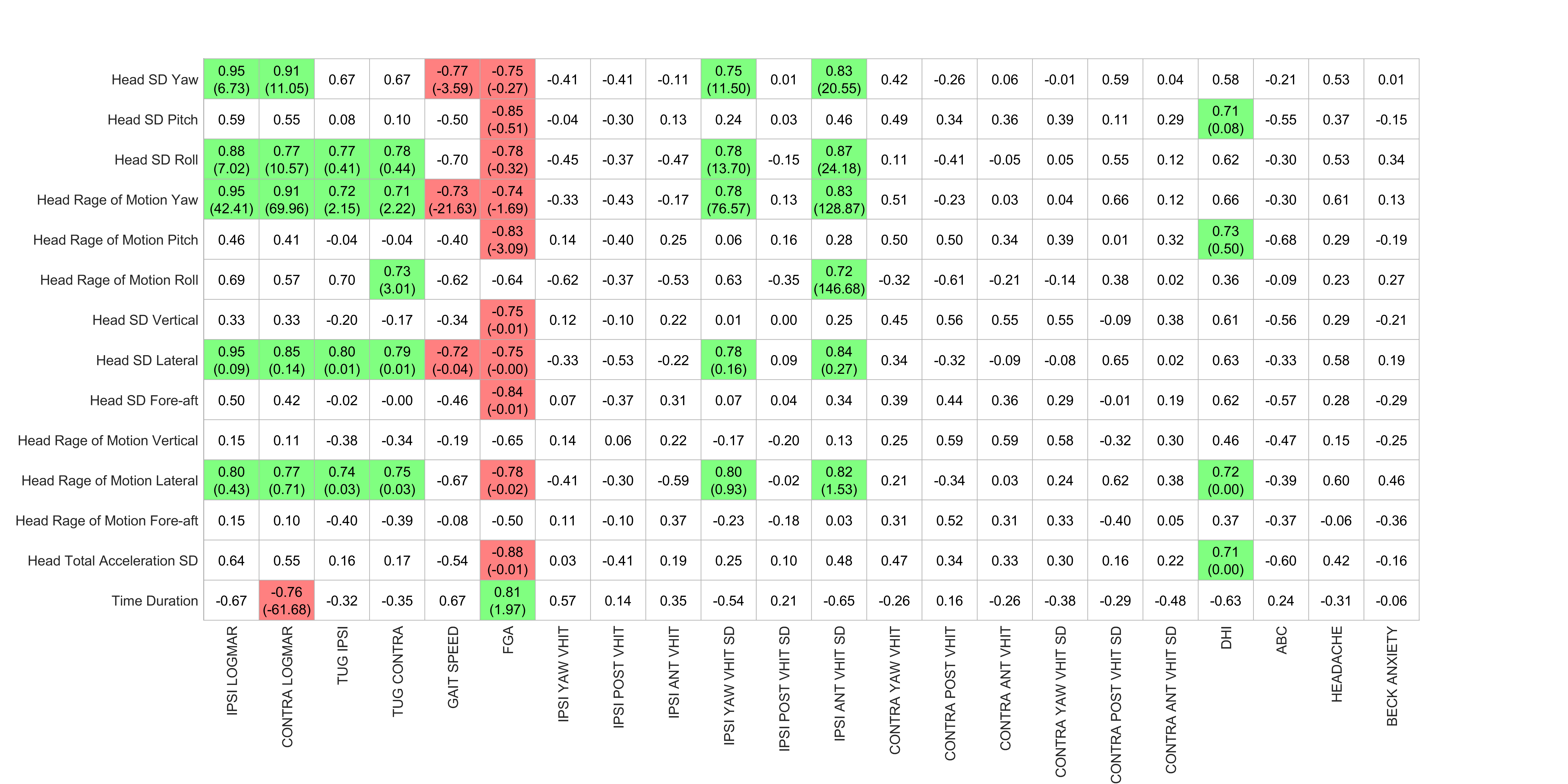


**Table 9- table supplement -** Correlation coefficients (slope) for Task “Standing on foam eyes closed” (Preop. Clinical vs. Preop. Kinematics)


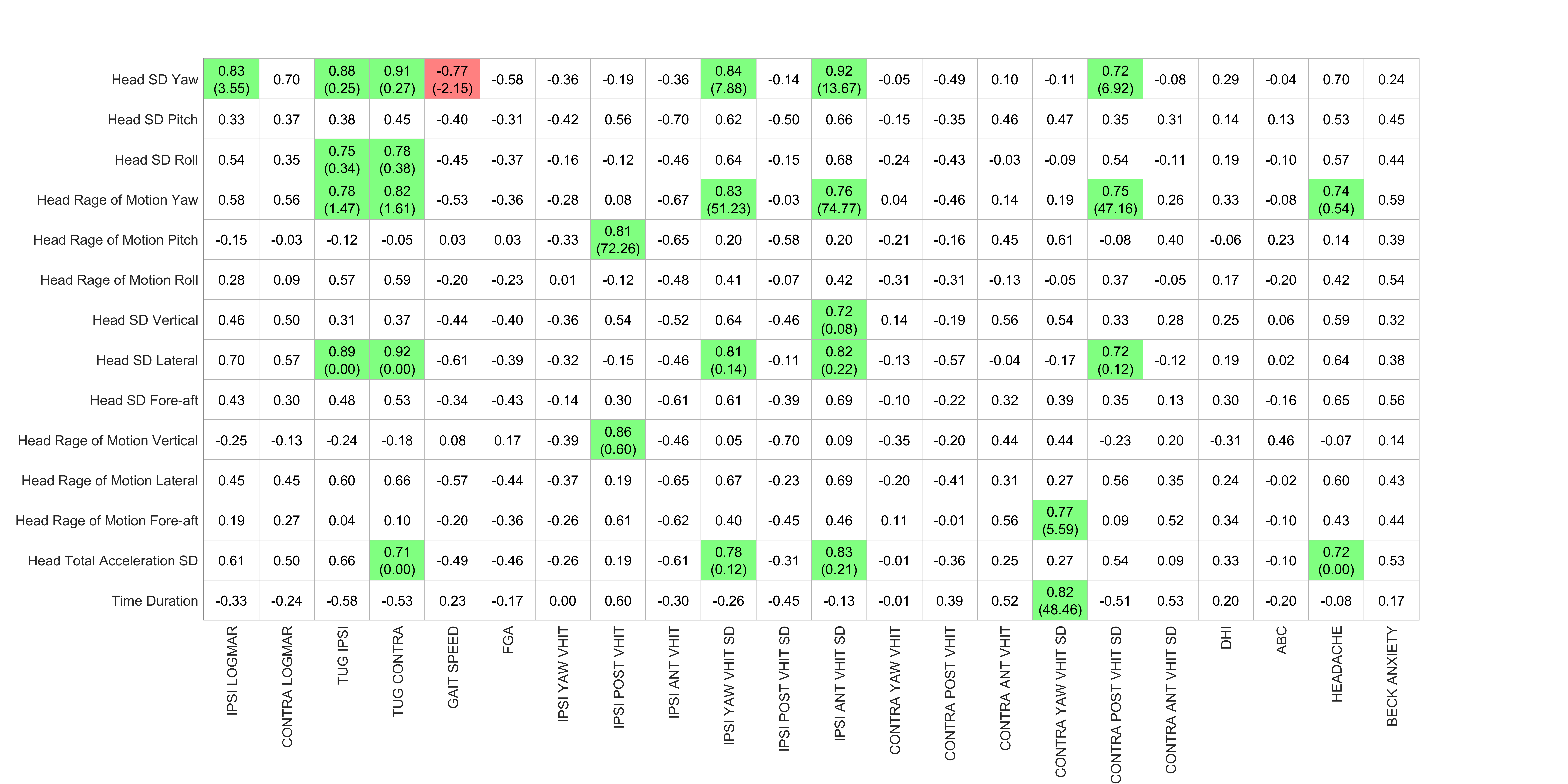


**Table 10- table supplement -** Correlation coefficients (slope) for Task “Standing on foam eyes open” (Preop. Clinical vs. Preop. Kinematics)


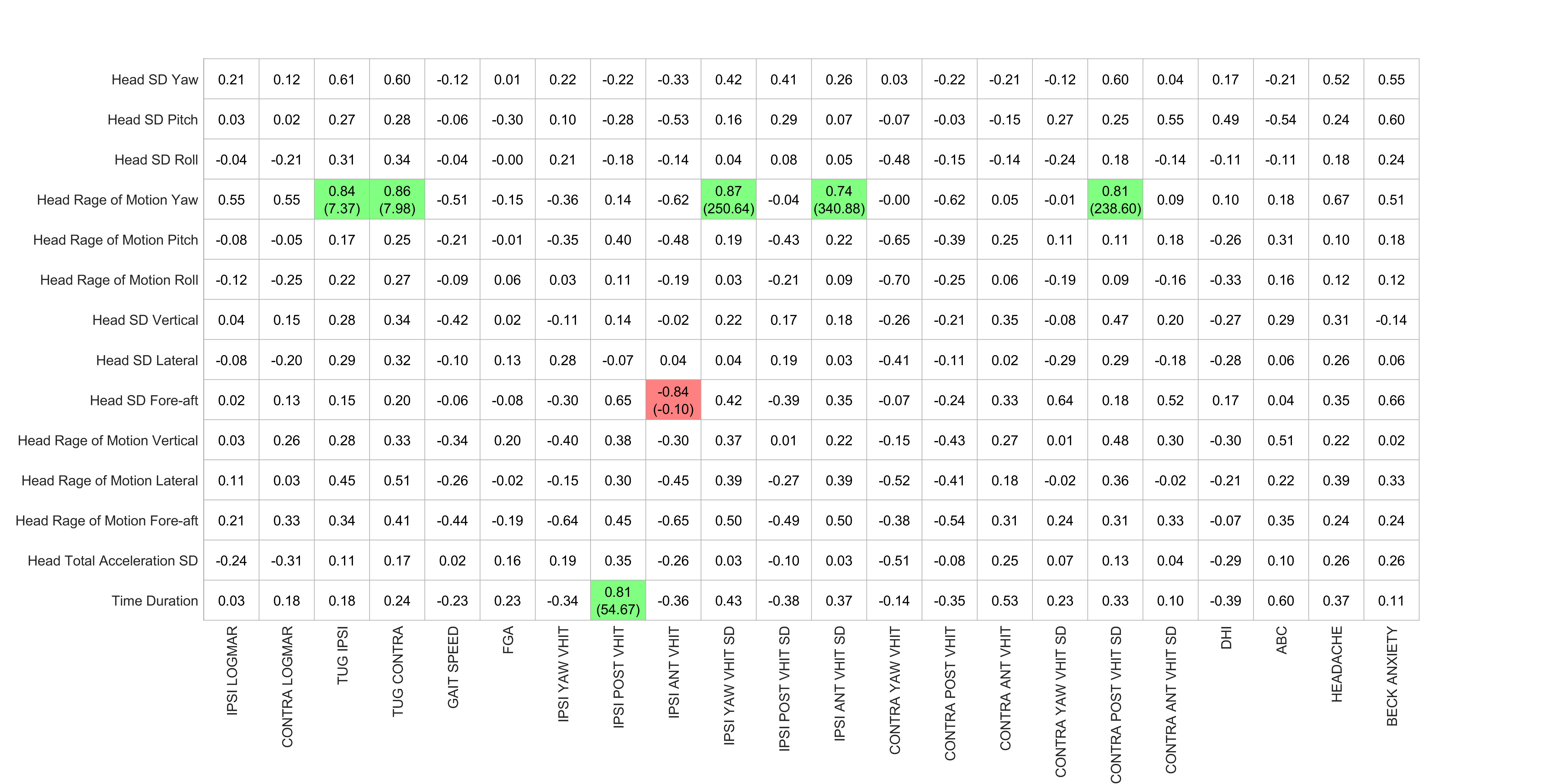


**Table 11- table supplement -** Correlation coefficients (slope) for Task “Foam cup balance 1 foot” (Preop. Clinical vs. Preop. Kinematics)


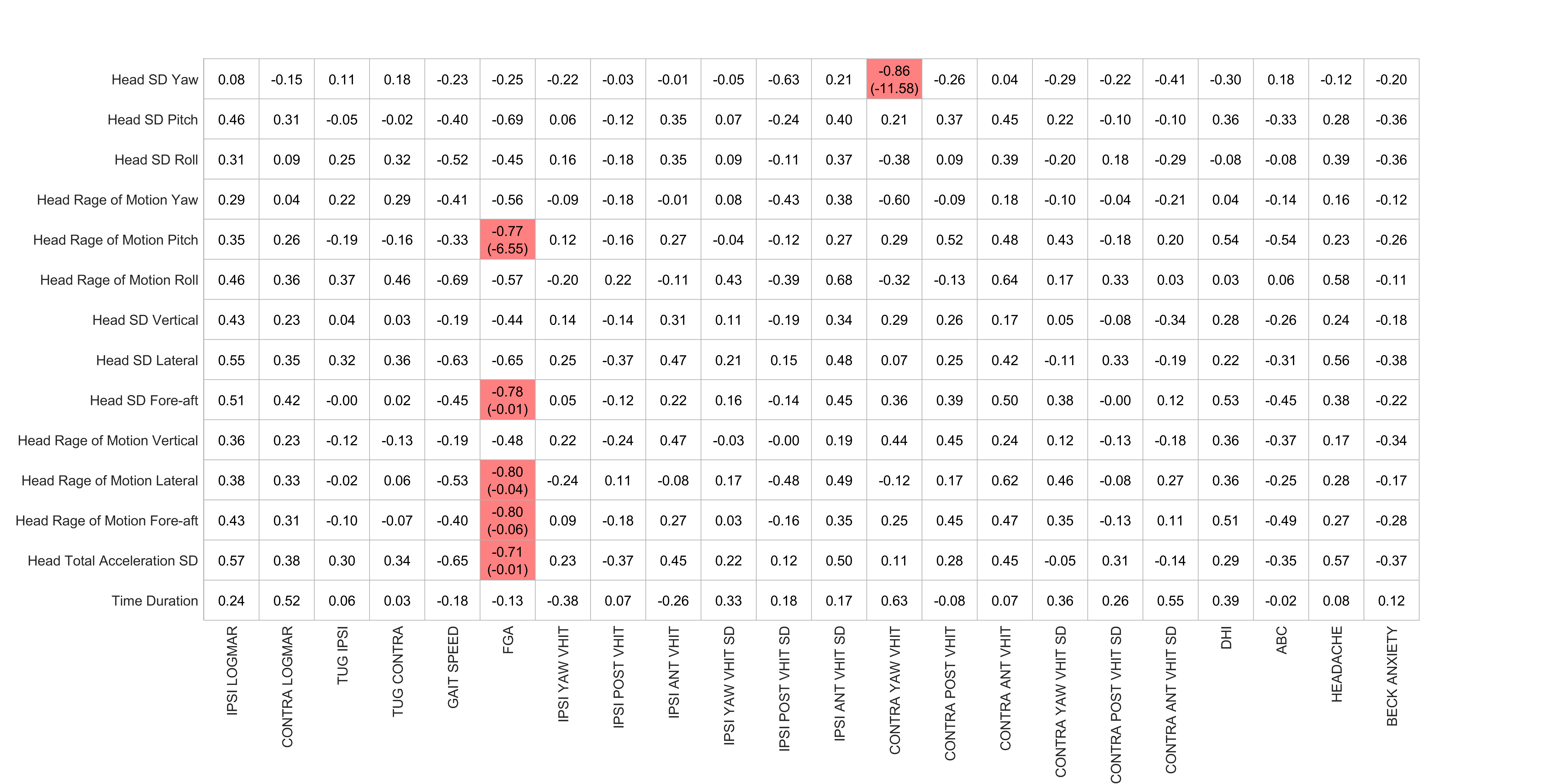


**Table 12- table supplement -** Correlation coefficients (slope) for Task “Foam cup alternatively foot” (Preop. Clinical vs. Preop. Kinematics)


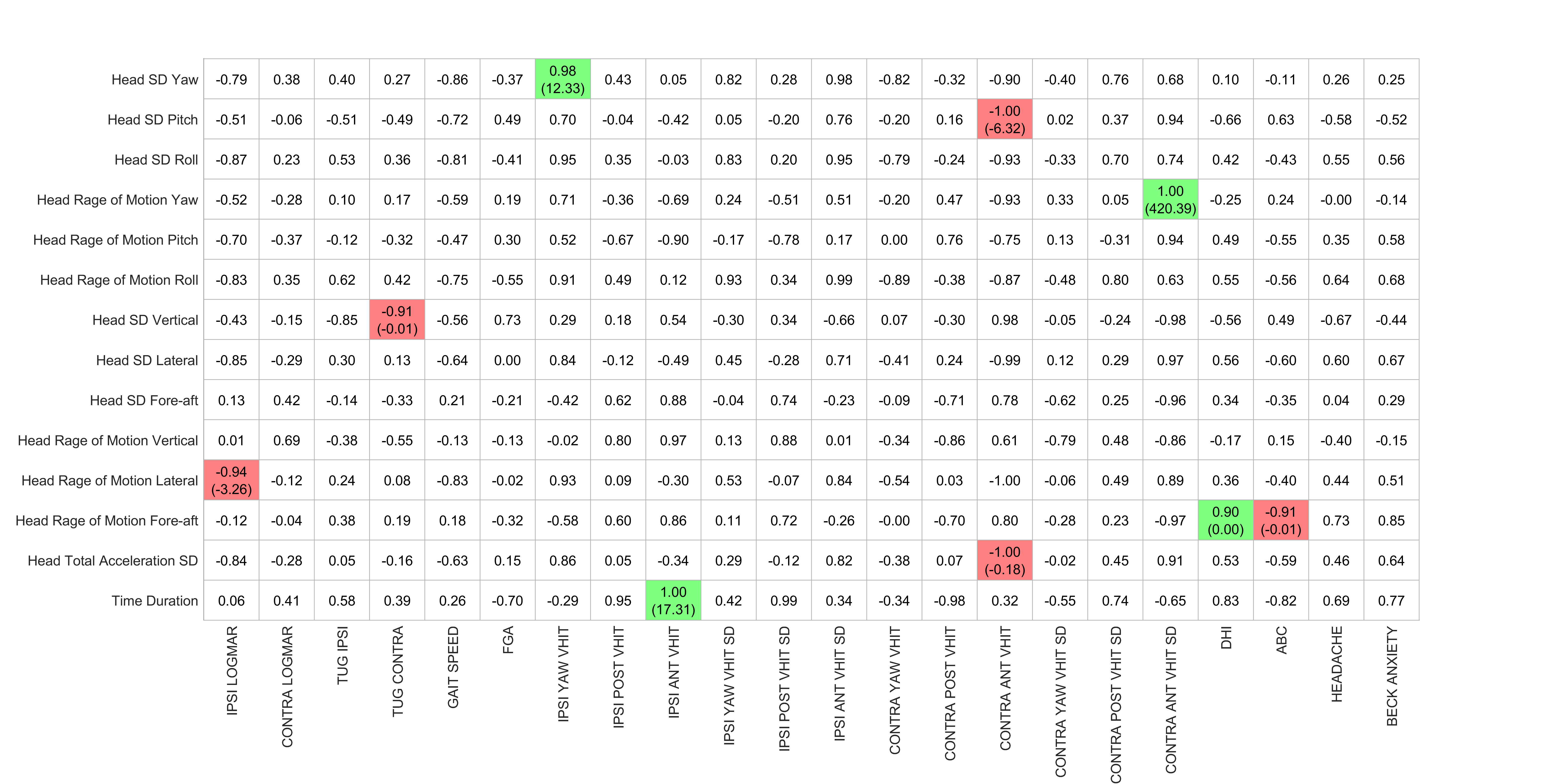


**Table 13- table supplement -** Correlation coefficients (slope) for Task “Tandem walk forward (FGA)” (Postop. Clinical vs. Postop. Kinematics)


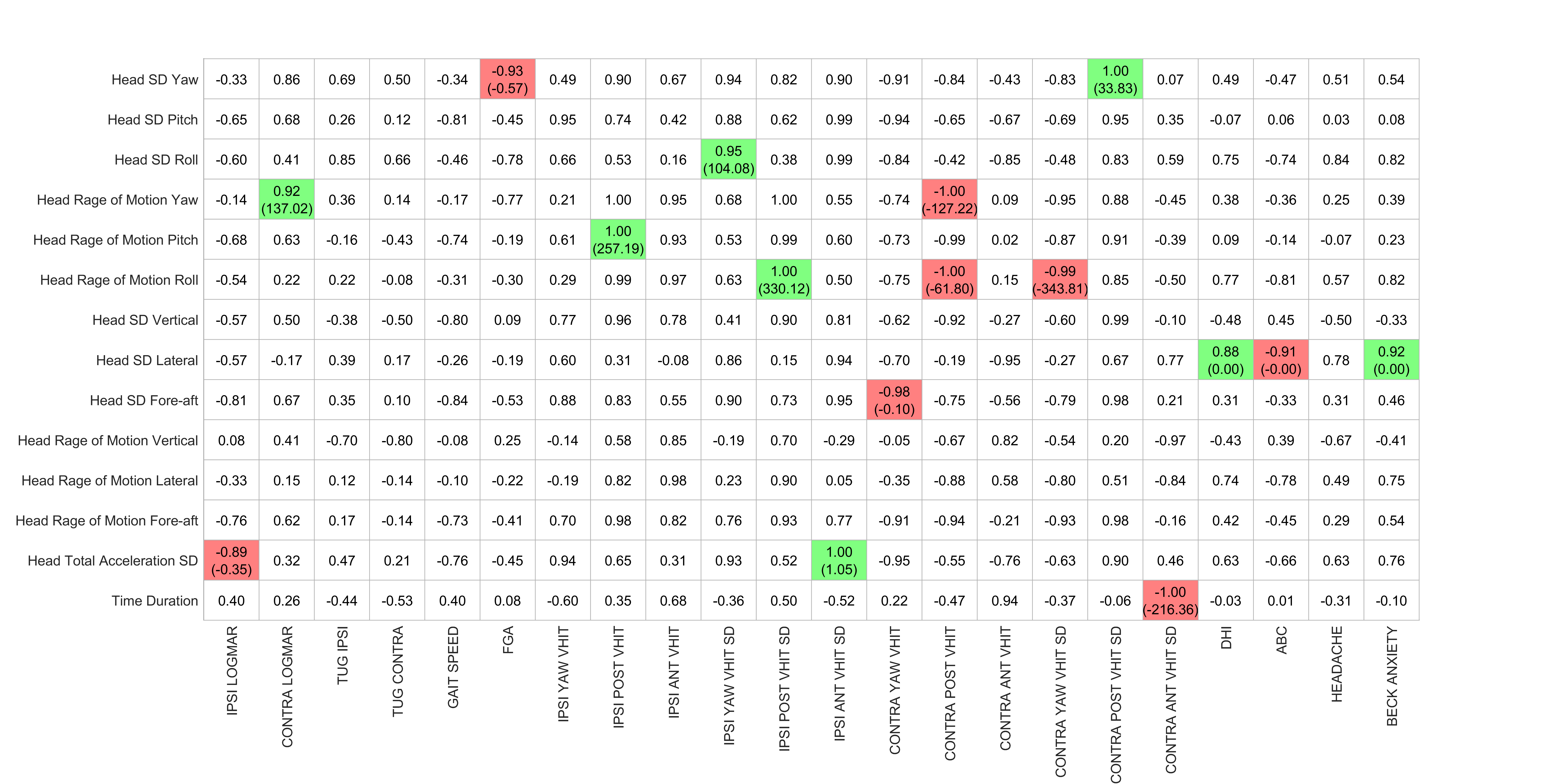


**Table 14- table supplement -** Correlation coefficients (slope) for Task “Tandem walk forward” (Postop. Clinical vs. Postop. Kinematics)


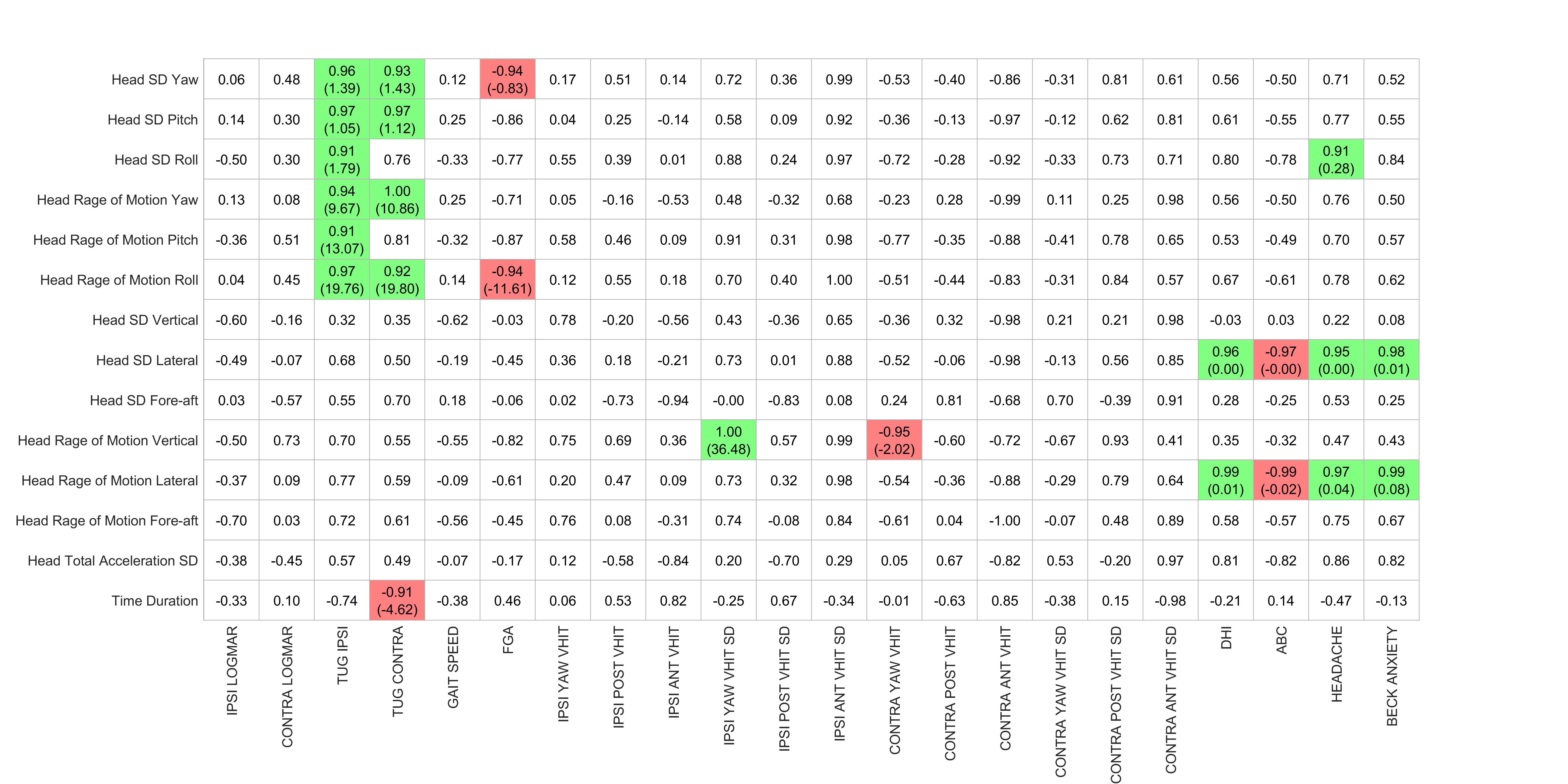


**Table 15- table supplement -** Correlation coefficients (slope) for Task “Tandem walk backward” (Postop. Clinical vs. Postop. Kinematics)


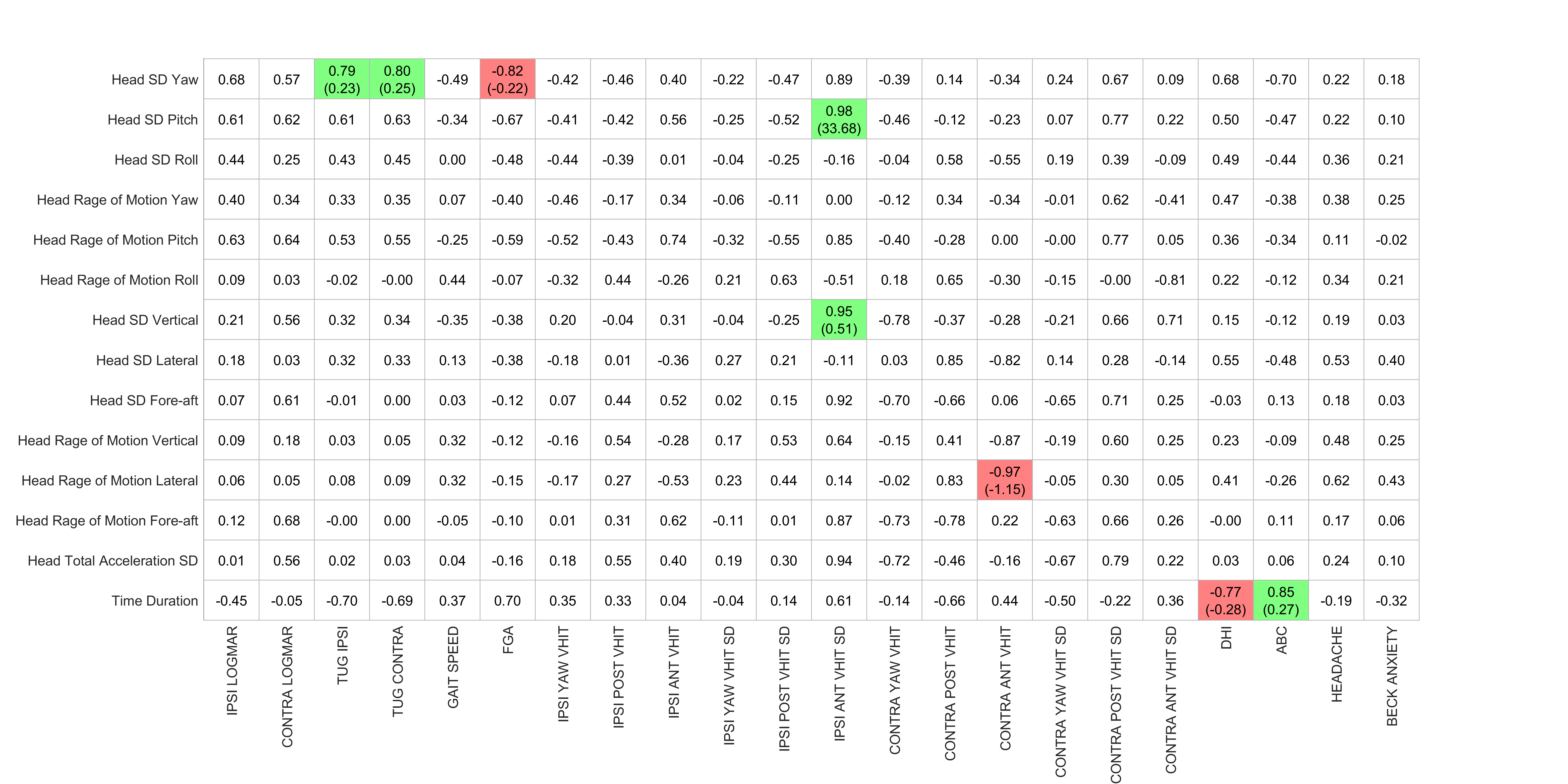


**Table 16- table supplement -** Correlation coefficients (slope) for Task “Tandem stance eyes open” (Postop. Clinical vs. Postop. Kinematics)


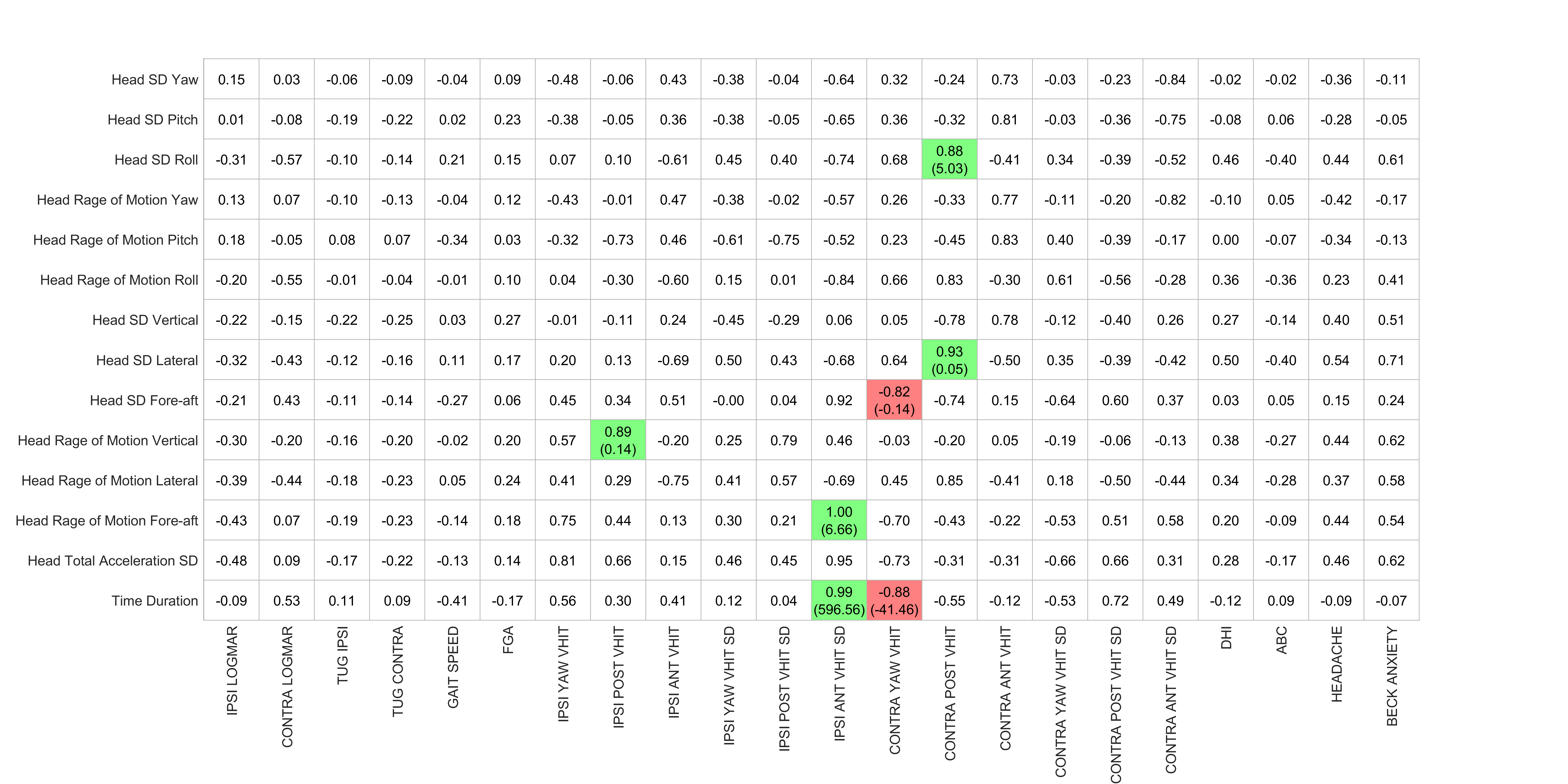


**Table 17- table supplement -** Correlation coefficients (slope) for Task “Tandem stance eyes closed” (Postop. Clinical vs. Postop. Kinematics)


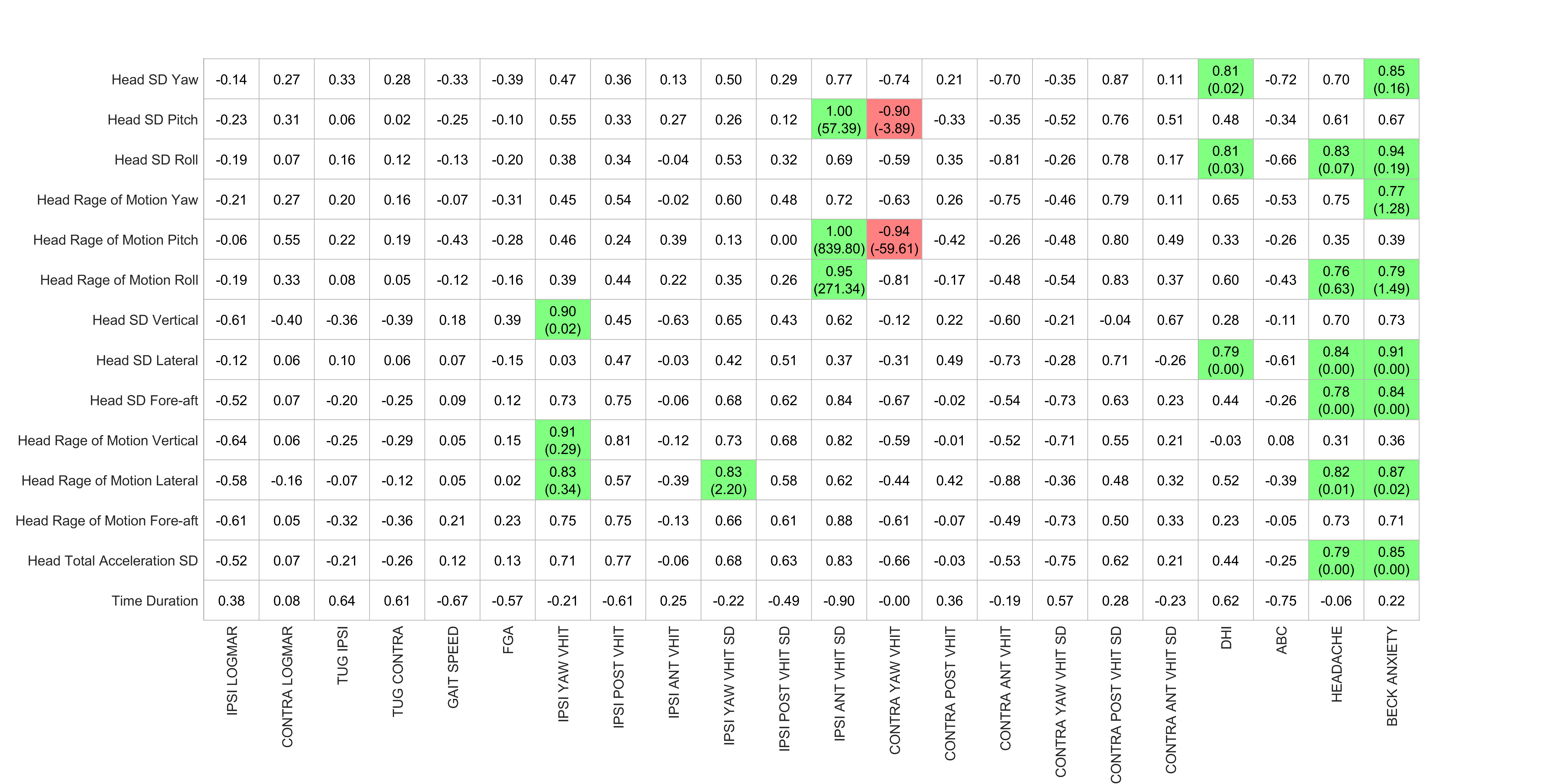


**Table 18- table supplement -** Correlation coefficients (slope) for Task “Standing on firm eyes closed” (Postop. Clinical vs. Postop. Kinematics)


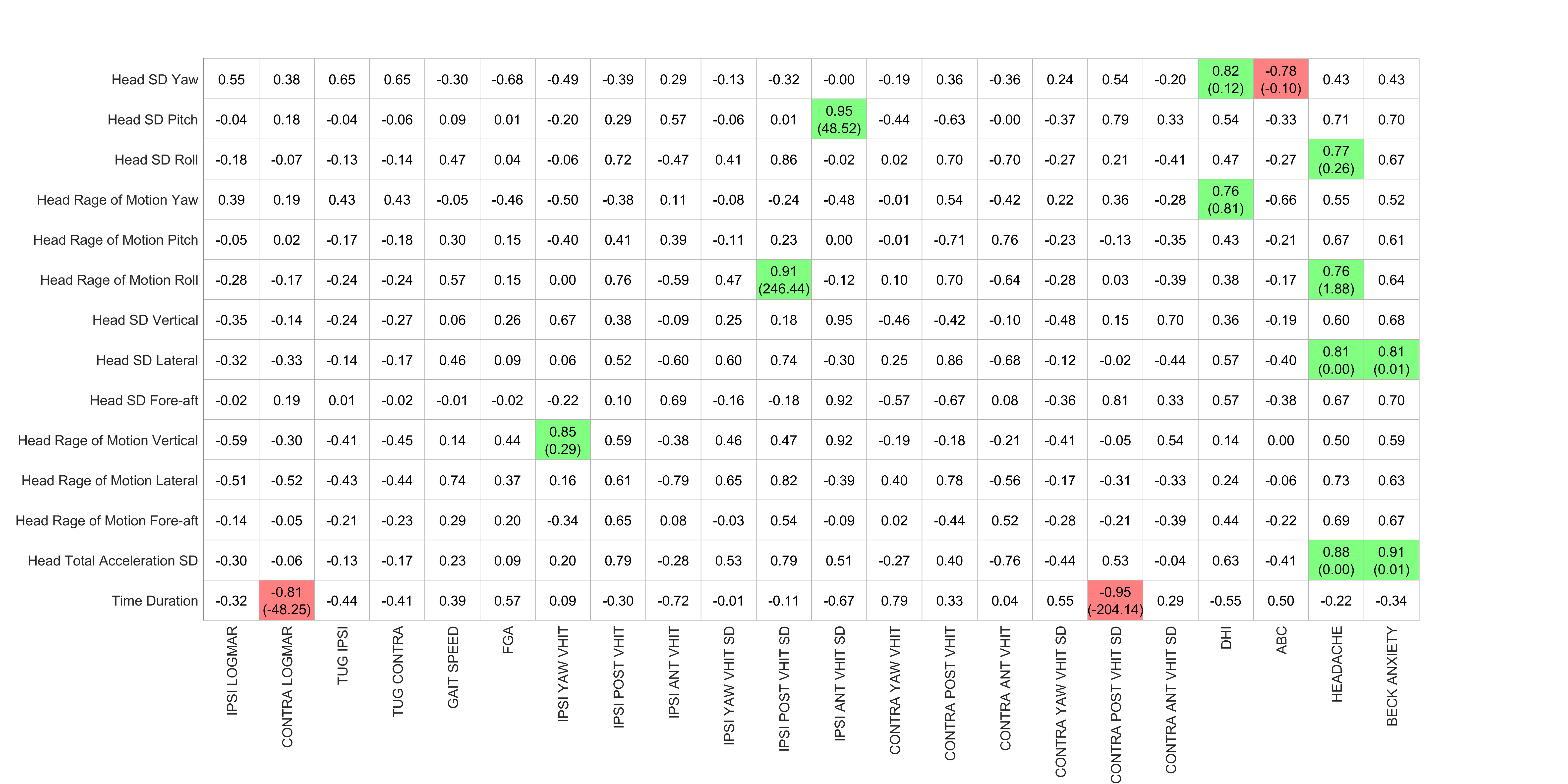


**Table 19- table supplement -** Correlation coefficients (slope) for Task “Standing on foam eyes closed” (Postop. Clinical vs. Postop. Kinematics)


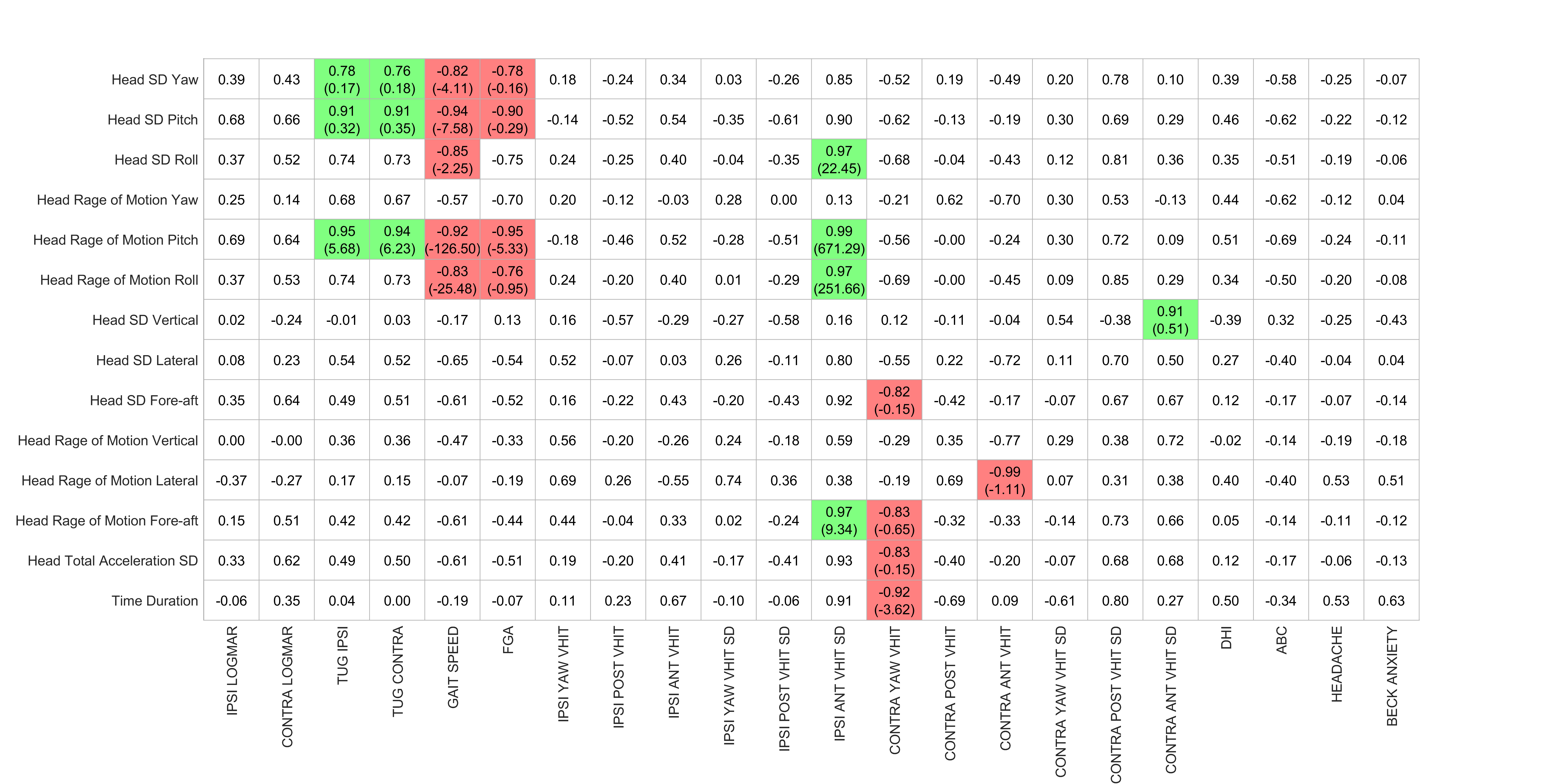


**Table 20- table supplement -** Correlation coefficients (slope) for Task “Standing on foam eyes open” (Postop. Clinical vs. Postop. Kinematics)


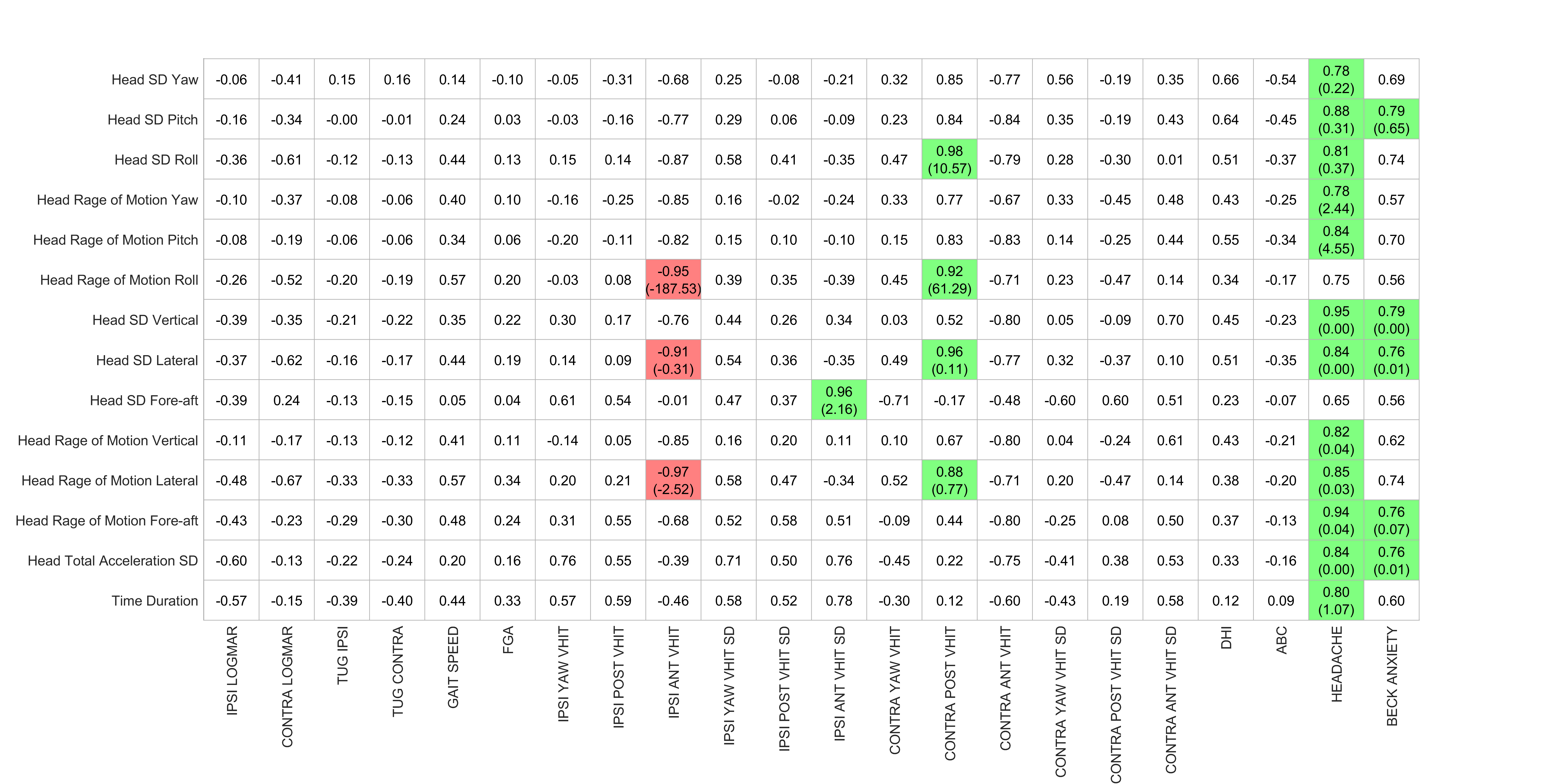


**Table 21- table supplement -** Correlation coefficients (slope) for Task “Foam cup balance 1 foot” (Postop. Clinical vs. Postop. Kinematics)

**
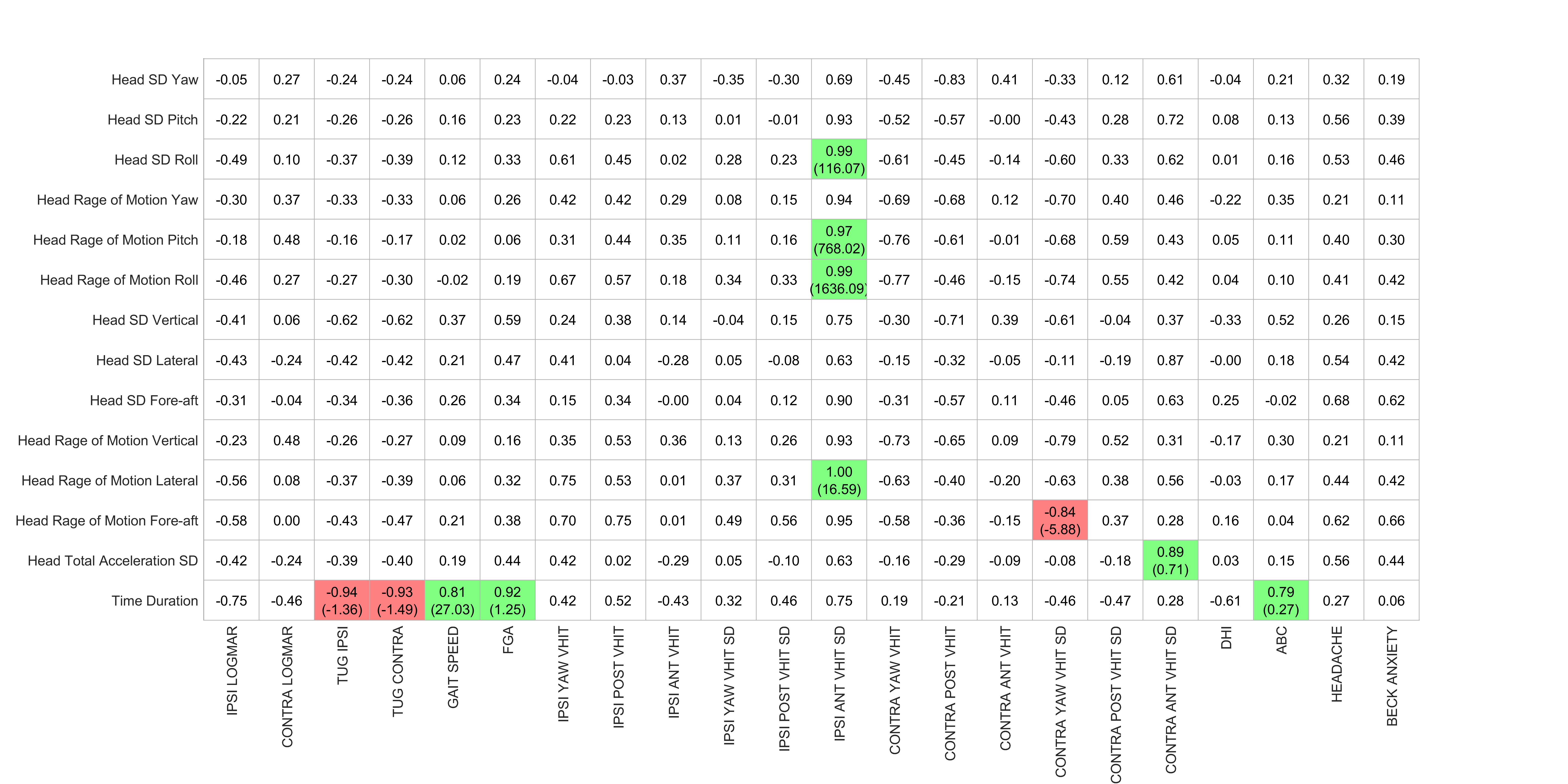
**

**Table 22- table supplement -** Correlation coefficients (slope) for Task “Foam cup alternatively foot” (Postop. Clinical vs. Postop. Kinematics)


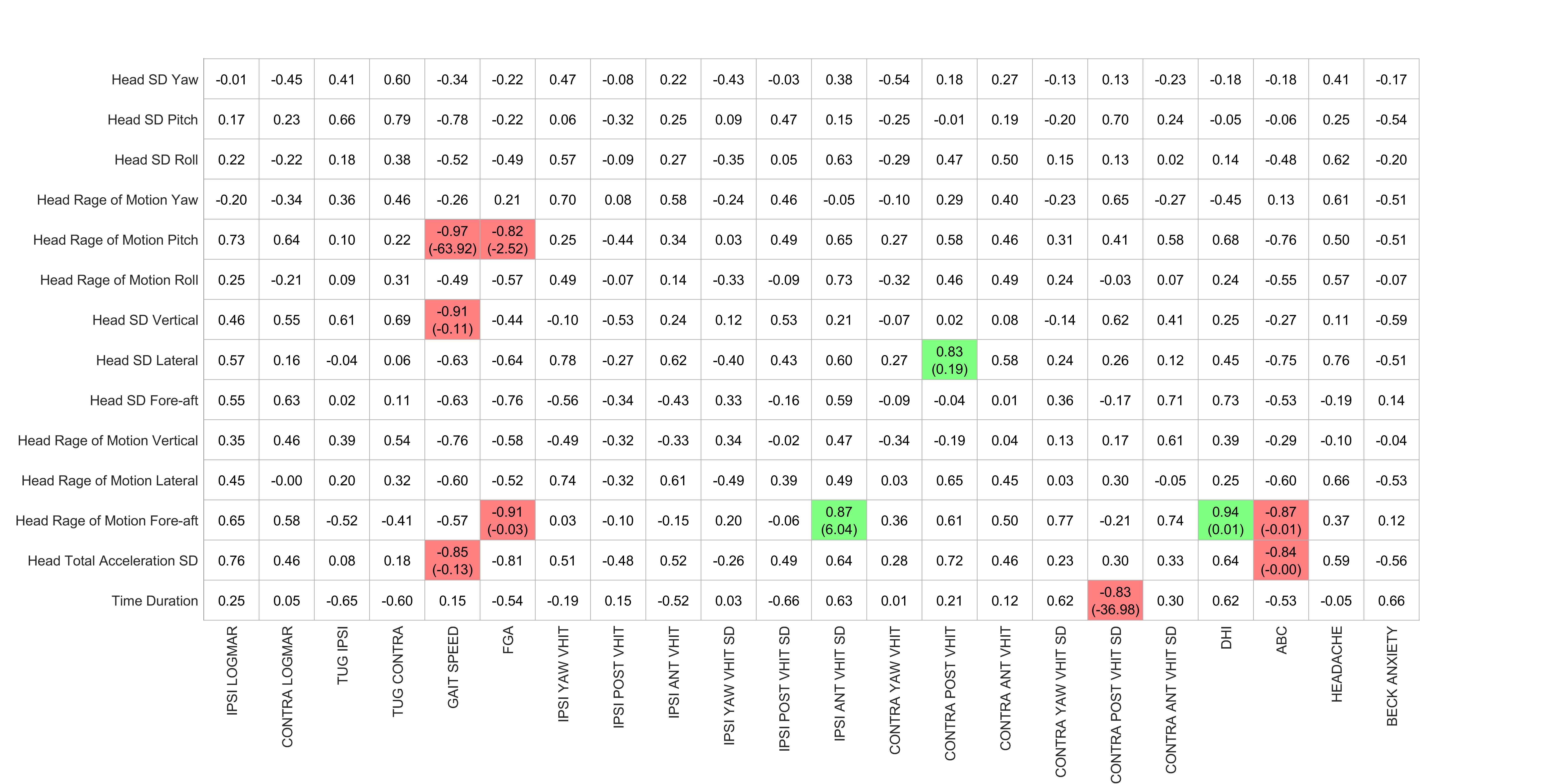


**Table 23- table supplement -** Correlation coefficients (slope) for Task “Tandem walk forward (FGA)” (Preop. Clinical vs. Postop. Kinematics)


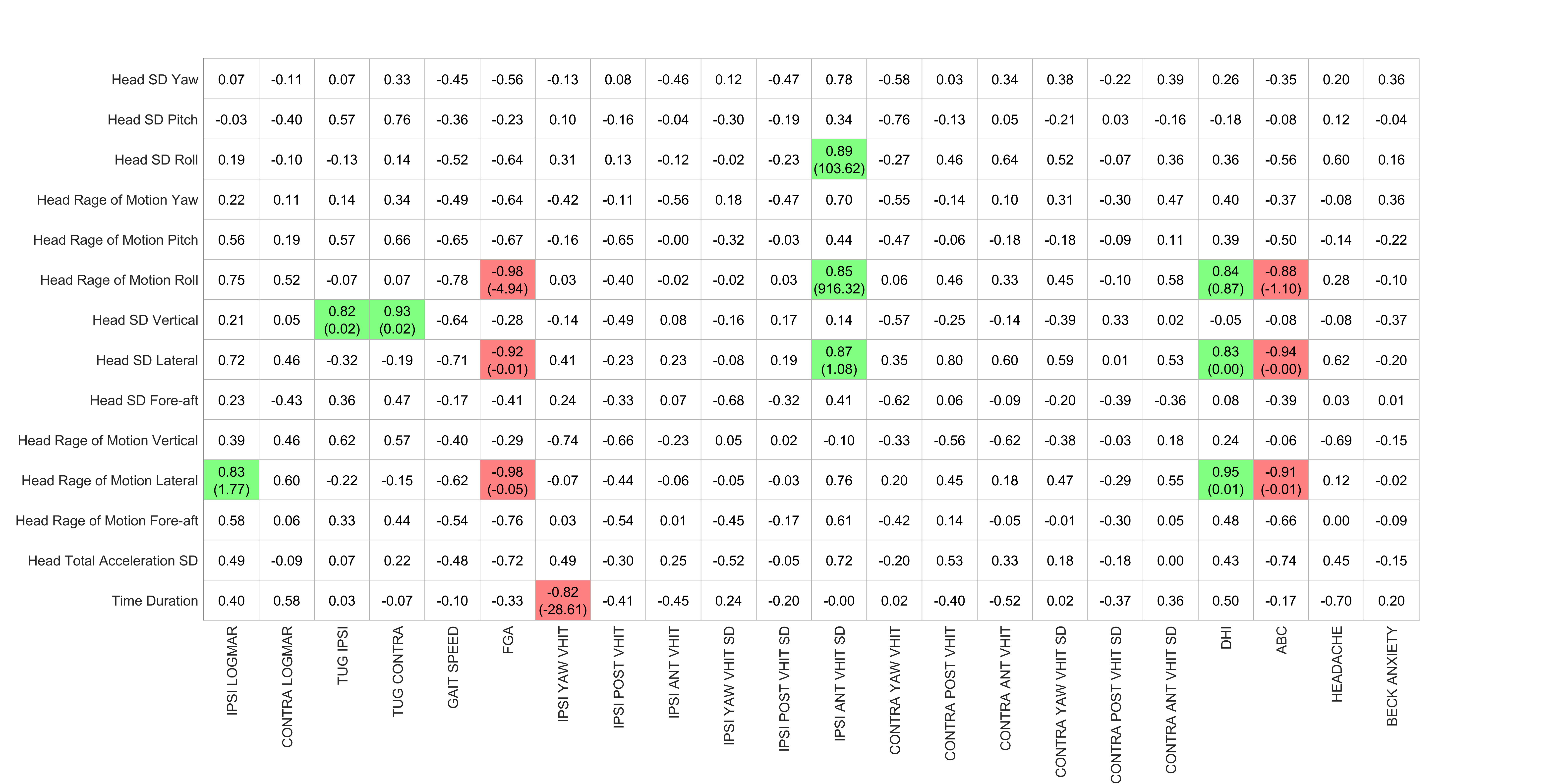


**Table 24- table supplement -** Correlation coefficients (slope) for Task “Tandem walk forward” (Preop. Clinical vs. Postop. Kinematics)


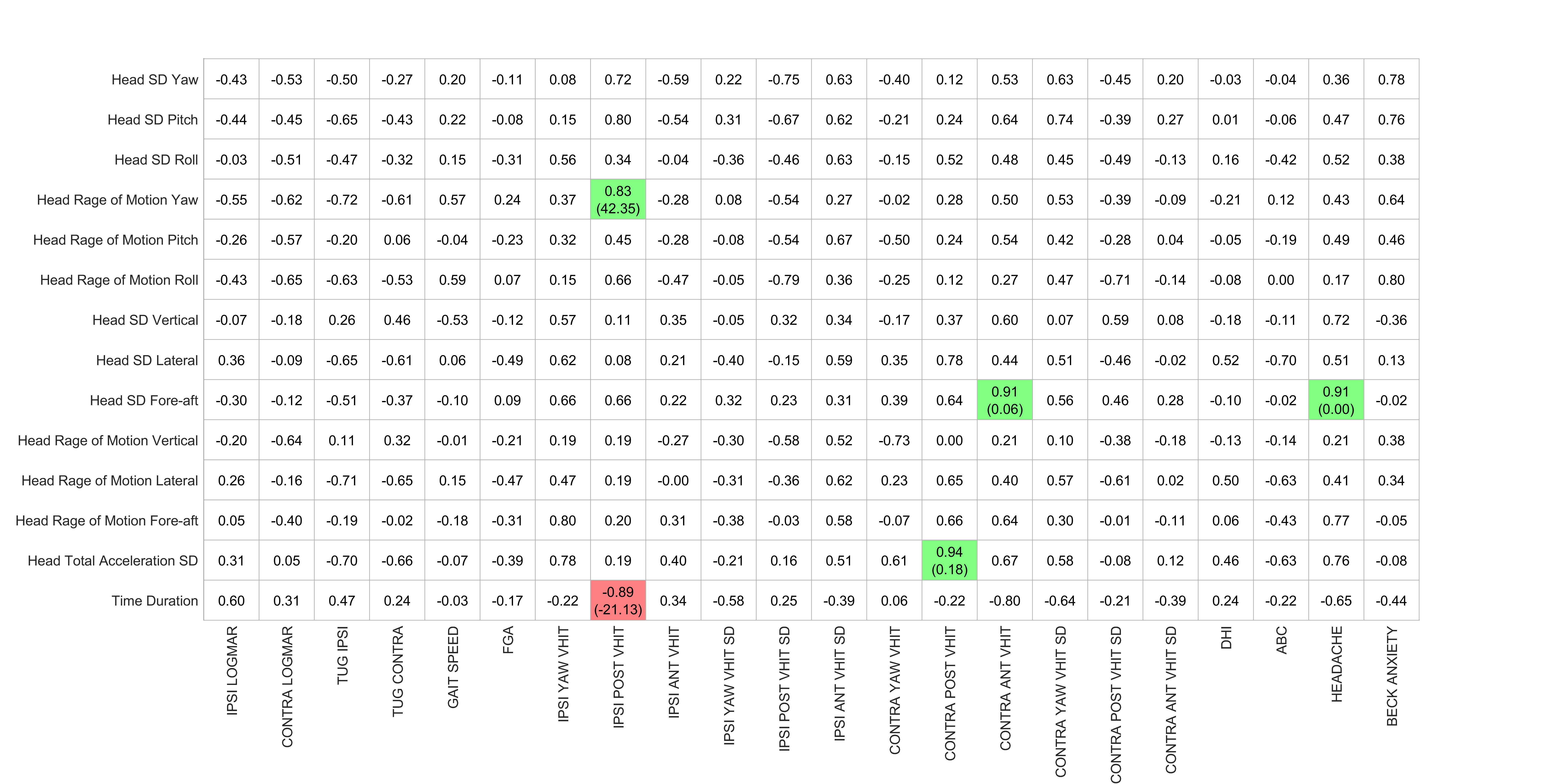


**Table 25- table supplement -** Correlation coefficients (slope) for Task “Tandem walk backward” (Preop. Clinical vs. Postop. Kinematics)


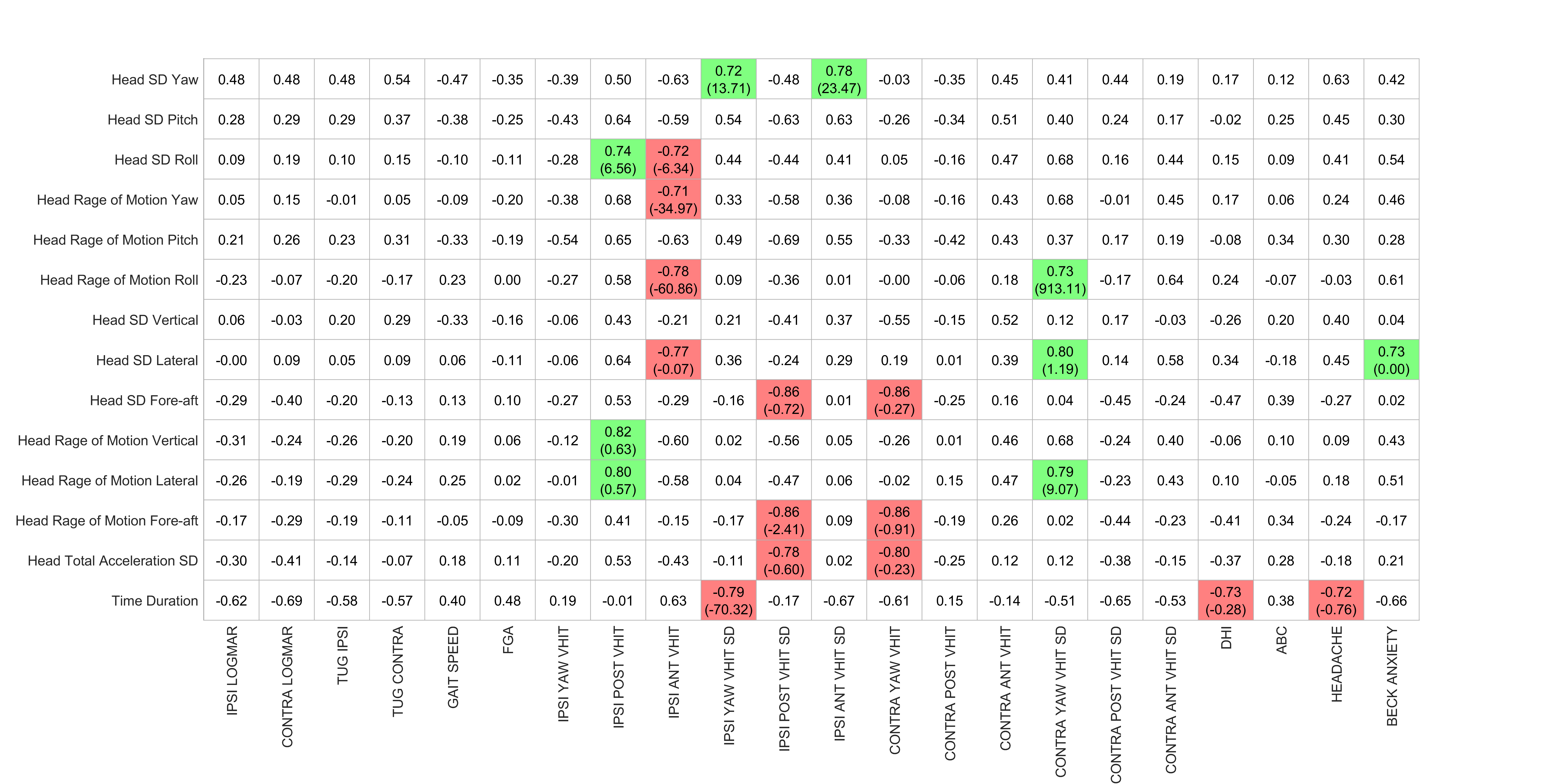


**Table 26- table supplement -** Correlation coefficients (slope) for Task “Tandem stance eyes open” (Preop. Clinical vs. Postop. Kinematics)


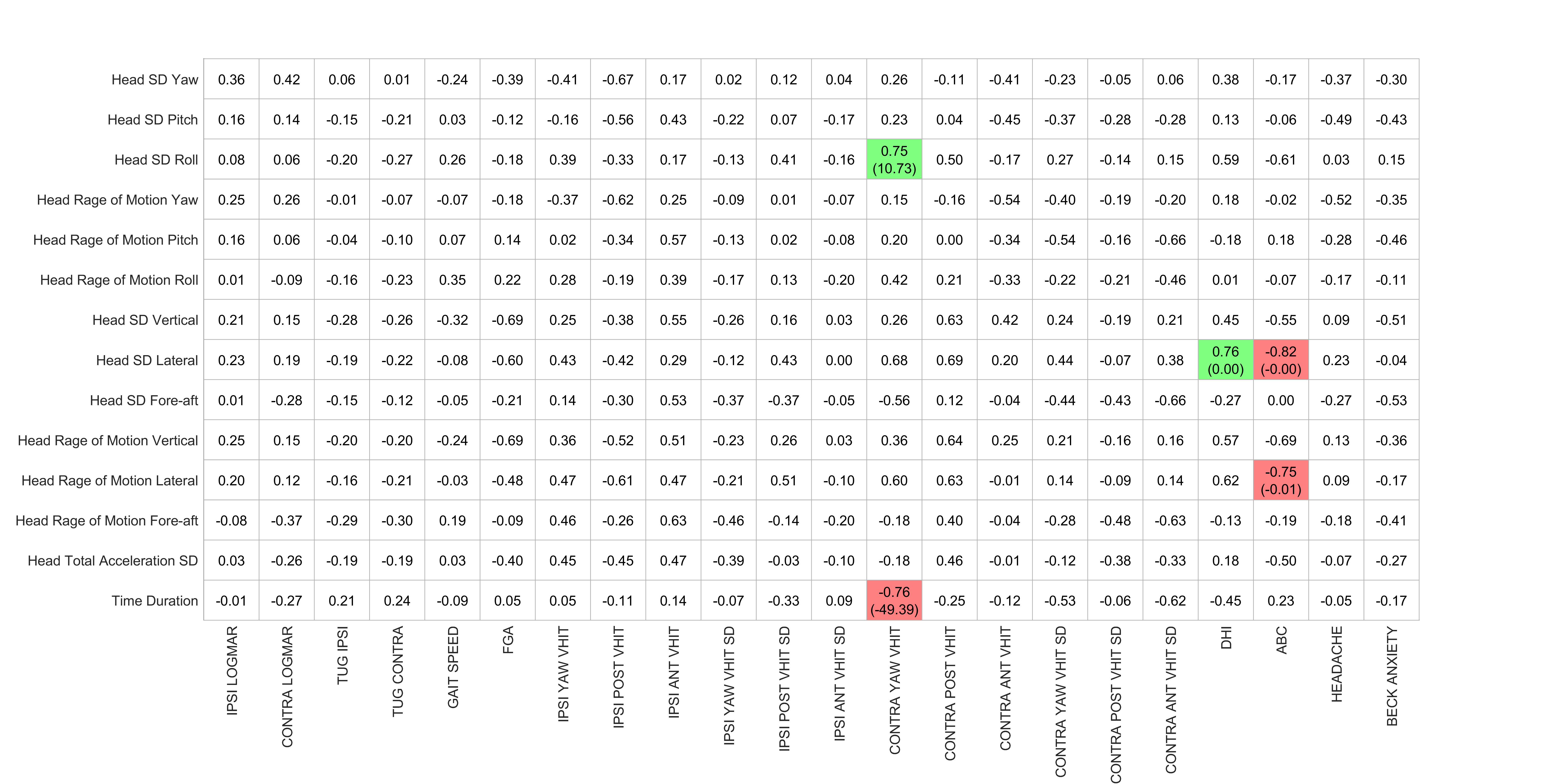


**Table 27- table supplement -** Correlation coefficients (slope) for Task “Tandem stance eyes closed” (Preop. Clinical vs. Postop. Kinematics)


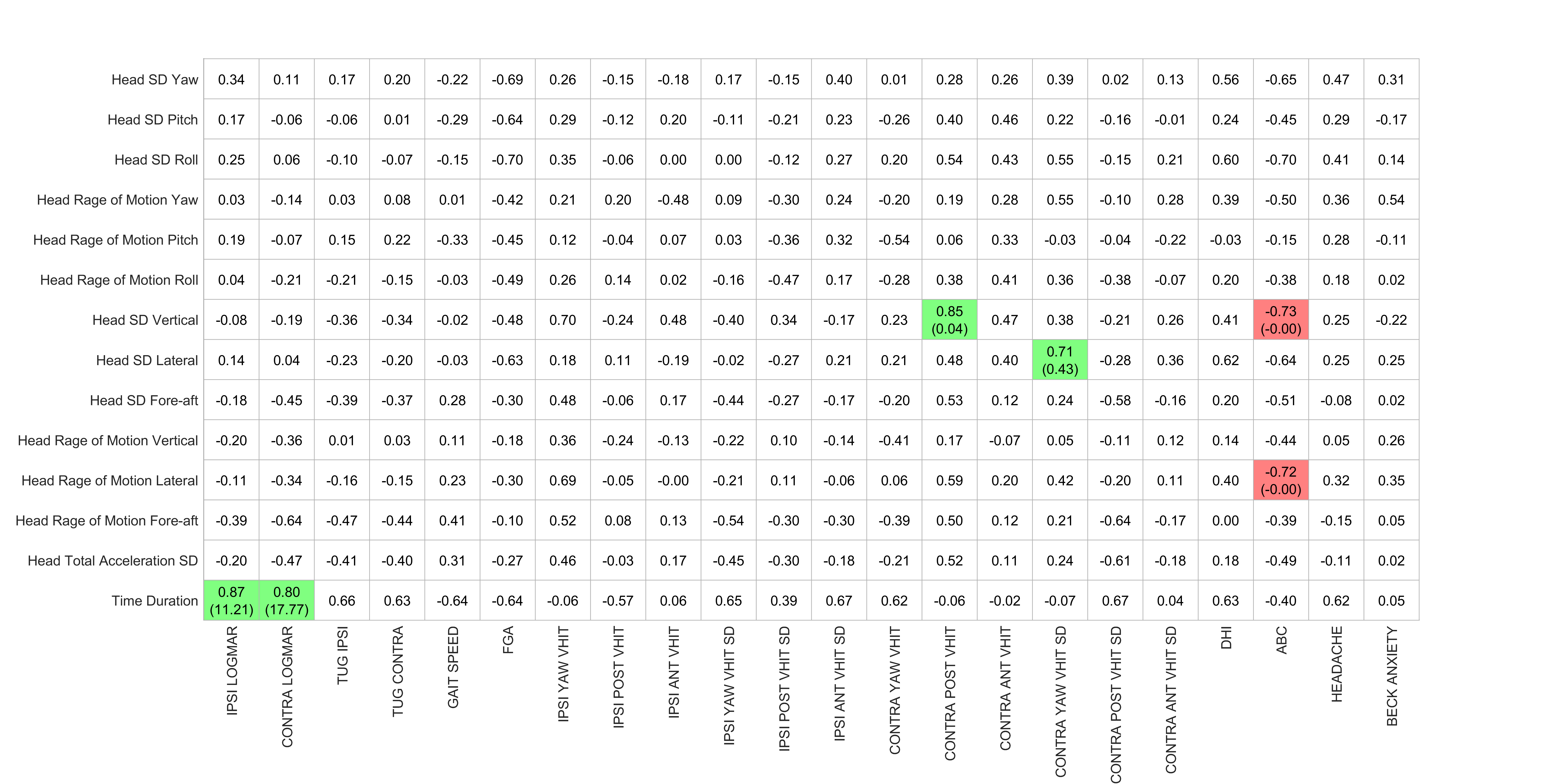


**Table 28- table supplement -** Correlation coefficients (slope) for Task “Standing on firm eyes closed” (Preop. Clinical vs. Postop. Kinematics)


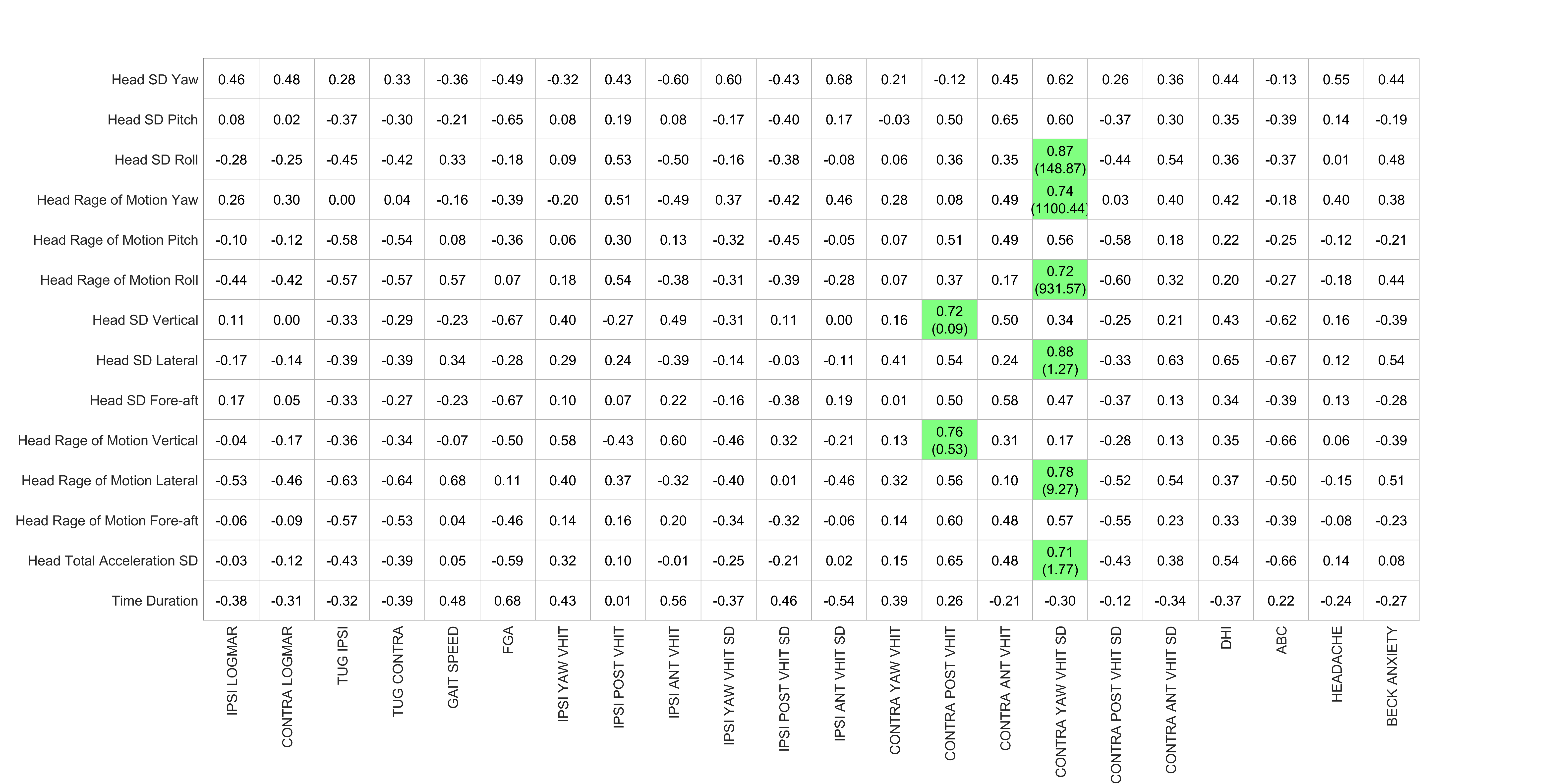


**Table 29- table supplement -** Correlation coefficients (slope) for Task “Standing on foam eyes closed” (Preop. Clinical vs. Postop. Kinematics)


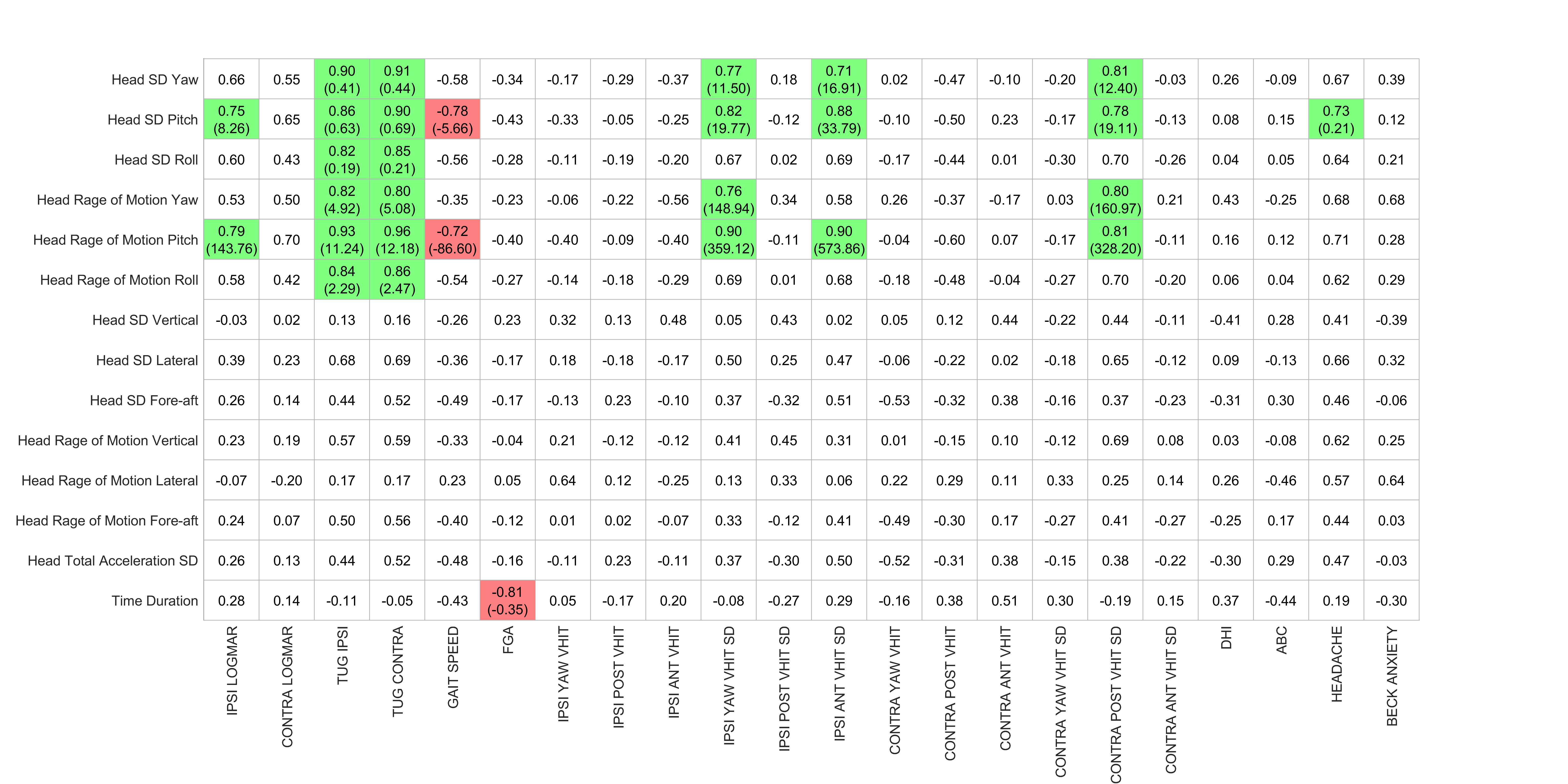


**Table 30- table supplement -** Correlation coefficients (slope) for Task “Standing on foam eyes open” (Preop. Clinical vs. Postop. Kinematics)


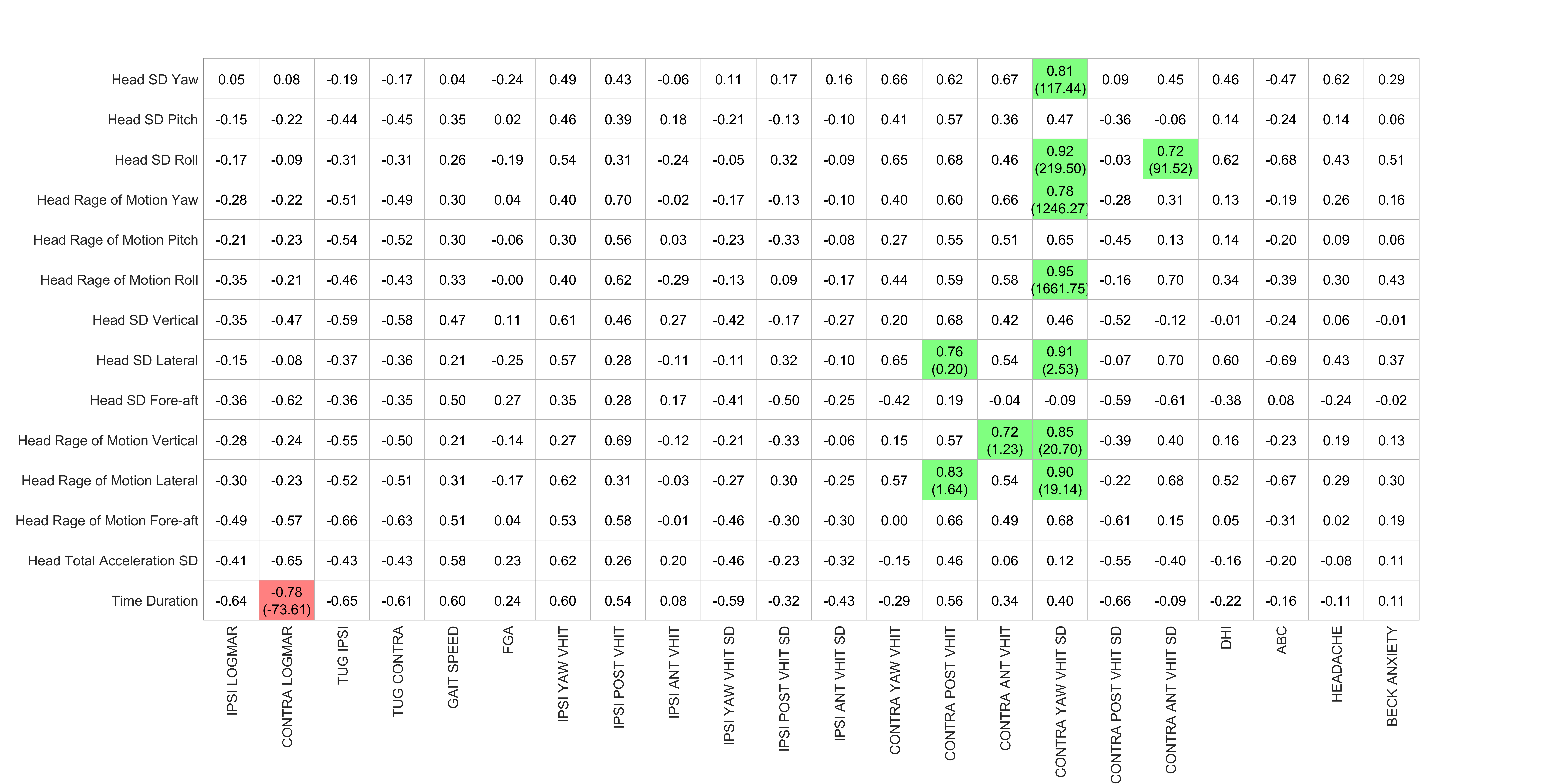


**Table 31- table supplement -** Correlation coefficients (slope) for Task “Foam cup balance 1 foot” (Preop. Clinical vs. Postop. Kinematics)


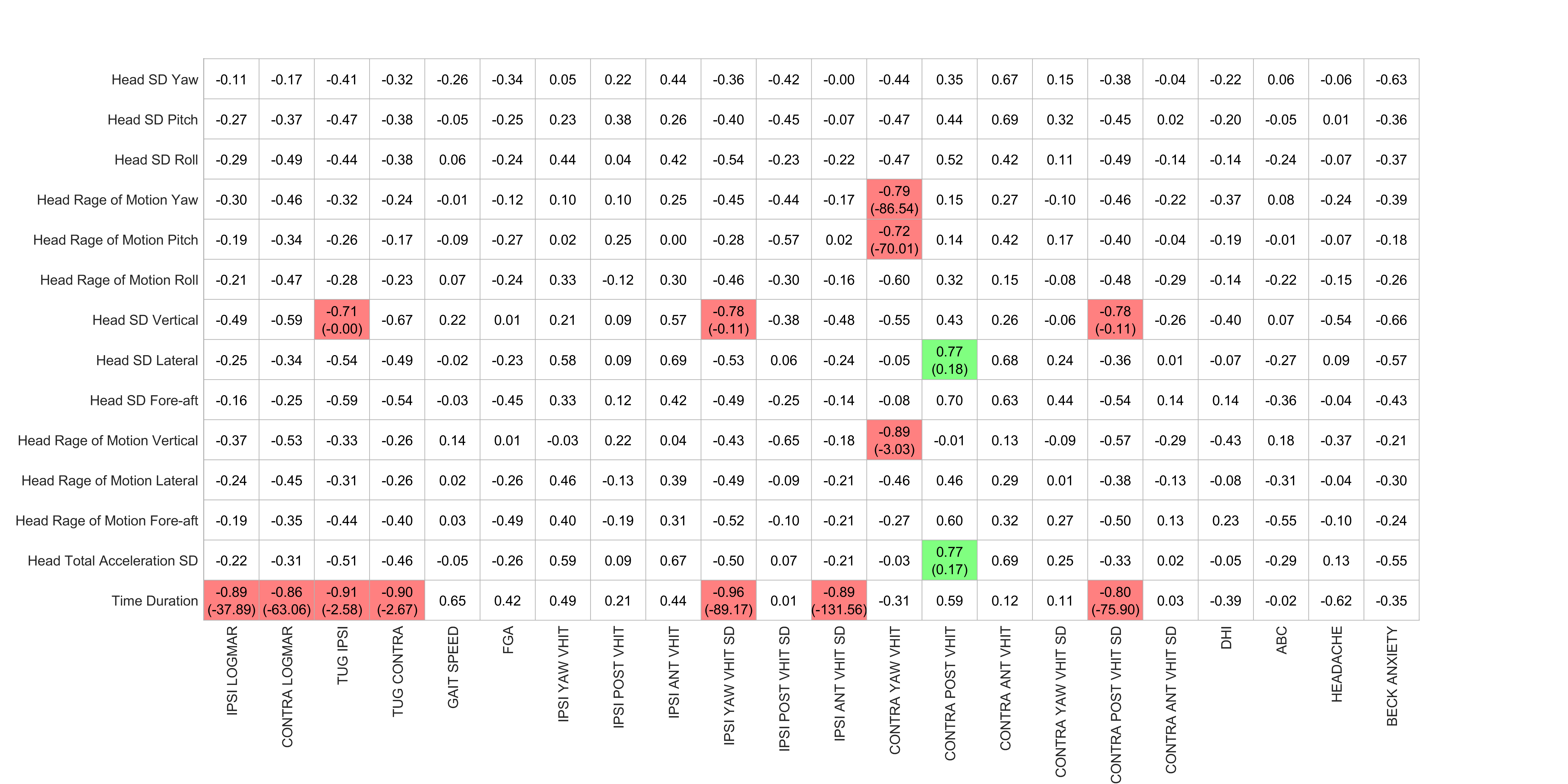


**Table 32- table supplement -** Correlation coefficients (slope) for Task “Foam cup alternatively foot” (Preop. Clinical vs. Postop. Kinematics)


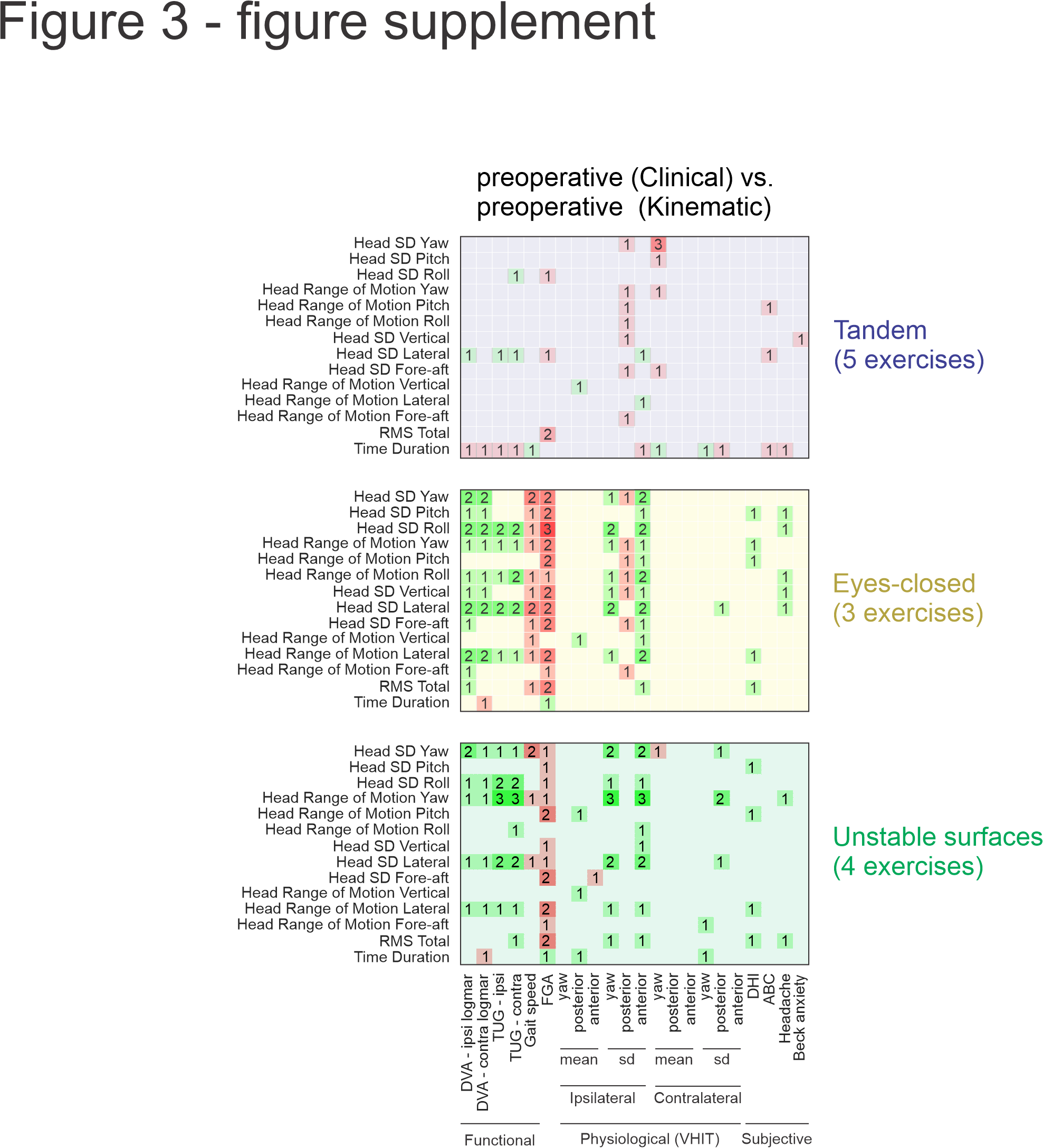


**Figure 3- figure supplement -** Correlation map between pre-surgery kinematic measurements and pre-surgery clinical measurements for top: tandem, middle: eyes-closed, and bottom: unstable surface balance exercises. Green squares indicate positive correlations and red squares indicate negative correlations. Brightness and number in the square indicate the number of exercises showing a significant correlation (p<0.05).
